# E2F1 K117 methylation by SETD6 disrupts BRD4–E2F1 binding and modulates E2F1 chromatin binding and gene regulation in prostate cancer cells

**DOI:** 10.1093/nar/gkaf1513

**Published:** 2026-01-15

**Authors:** Gizem Tugce Ulu, Margarita Kublanovsky, Raz Shalev, Tzofit Elbaz Biton, Michal Feldman, Sophia Murr, Jens Brockmeyer, Franziska Dorscht, Sara Weirich, Dan Levy, Albert Jeltsch

**Affiliations:** Institute of Biochemistry, University of Stuttgart, Allmandring 31, Stuttgart 70569, Germany; The Shraga Segal Department of Microbiology, Immunology and Genetics, Ben-Gurion University of the Negev, P.O.B. 653, Be’er-Sheva 84105, Israel; The National Institute for Biotechnology in the Negev, Ben-Gurion University of the Negev, P.O.B. 653, Be’er-Sheva 84105, Israel; The Shraga Segal Department of Microbiology, Immunology and Genetics, Ben-Gurion University of the Negev, P.O.B. 653, Be’er-Sheva 84105, Israel; The National Institute for Biotechnology in the Negev, Ben-Gurion University of the Negev, P.O.B. 653, Be’er-Sheva 84105, Israel; The Shraga Segal Department of Microbiology, Immunology and Genetics, Ben-Gurion University of the Negev, P.O.B. 653, Be’er-Sheva 84105, Israel; The National Institute for Biotechnology in the Negev, Ben-Gurion University of the Negev, P.O.B. 653, Be’er-Sheva 84105, Israel; The Shraga Segal Department of Microbiology, Immunology and Genetics, Ben-Gurion University of the Negev, P.O.B. 653, Be’er-Sheva 84105, Israel; The National Institute for Biotechnology in the Negev, Ben-Gurion University of the Negev, P.O.B. 653, Be’er-Sheva 84105, Israel; Institute of Biochemistry, University of Stuttgart, Allmandring 31, Stuttgart 70569, Germany; Institute of Biochemistry, University of Stuttgart, Allmandring 31, Stuttgart 70569, Germany; Institute of Biochemistry, University of Stuttgart, Allmandring 31, Stuttgart 70569, Germany; Institute of Biochemistry, University of Stuttgart, Allmandring 31, Stuttgart 70569, Germany; The Shraga Segal Department of Microbiology, Immunology and Genetics, Ben-Gurion University of the Negev, P.O.B. 653, Be’er-Sheva 84105, Israel; The National Institute for Biotechnology in the Negev, Ben-Gurion University of the Negev, P.O.B. 653, Be’er-Sheva 84105, Israel; Institute of Biochemistry, University of Stuttgart, Allmandring 31, Stuttgart 70569, Germany

## Abstract

The SETD6 (SET domain-containing protein 6) protein lysine methyltransferase regulates various cellular processes including cancer initiation and progression. It monomethylates the transcription factor E2F1 (E2F transcription factor 1) and several other important proteins, but the functional consequences of many SETD6 mediated methylation events are unknown. In this study, the role of SETD6 mediated K117 monomethylation of E2F1 was investigated in prostate cancer cells. In chromatin binding and gene expression experiments, we identified distinct sets of genes that are bound and upregulated by methylated and unmethylated E2F1 indicating that E2F1 methylation by SETD6 directly modulates its chromatin interaction. In agreement with these findings, cellular data showed that E2F1 methylation affects oncogenic phenotypes. Mechanistically, we demonstrate with biochemical, cellular, and genomic assays that SETD6-mediated K117 methylation directly regulates the interaction of E2F1 and BRD4 by preventing K117 acetylation. Our data suggest that K117 methylation/acetylation represents a switch controlling bromodomain binding to E2F1 by which SETD6 methylation regulates different cellular effects of E2F1. Similar mechanisms may apply to the regulation of other transcription factors by SETD6.

## Graphical Abstract

**Graphical Abstract:**
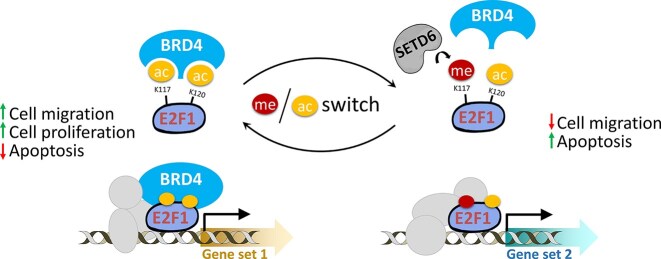

## Introduction

Transcription factors have a key role in regulating cellular functions related to diverse cell processes such as survival, growth, proliferation, metabolism, and cell death pathways [1–3]. The post-translational modification (PTM) of transcription factors can cause multiple changes of their activities, including changes in protein activity, protein stability, and protein–protein interactions that influence transcriptional and translational processes [4–6]. Hence, investigating the role of PTMs in transcription factors is critical to understand the dynamic functional regulation of gene expression. Lysine methylation is a common protein modification that is catalyzed by protein lysine methyltransferases (PKMTs) [7]. One enzyme of this class is SET domain-containing protein 6 (SETD6) that consists of a catalytic SET domain and a Rubisco substrate-binding domain [8, 9]. SETD6 is known as lysine mono-methyltransferase and it catalyzes lysine methylation of various histone and non-histone target proteins, for example, NF-κB subunit RelA K310 [10], K473 of the PAK4 kinase [11], K209 of PLK1 kinase [12], K33 of the TWIST1 transcription factor [13], K99 of the transcriptional coactivator BRD4 [14], and K12 of histone H4 [15, 16]. In many of these cases, SETD6 methylation was demonstrated to have pronounced effects on the cellular activities of the target proteins showing that SETD6 regulates different cellular processes in normal cells and various types of cancer.

The E2F transcription factor 1 (E2F1) regulates the expression of genes involved in different cellular processes in cancer including cell cycle, cell proliferation, apoptosis, and cellular metabolism [17–20]. E2F1 has been shown to be acetylated by PCAF/KAT2B at K117 and K120 and these modifications are associated with DNA damage [21, 22]. Acetylation of E2F1 creates binding sites for Bromodomain (BD) containing proteins [23] including BRD4 that was shown to bind to E2F1 in its diacetylated form with its first BD (BD1) [24]. In our previous work, we demonstrated that SETD6 methylates E2F1 at K117 [16, 25] and that this methylation positively regulates the expression of SETD6 in a positive feedback mechanism [25]. However, further functional effects of E2F1 K117 methylation by SETD6 are not known. This study investigates E2F1 chromatin interaction and gene regulation as well as the E2F1–BRD4 interaction in SETD6 wild type (WT) and SETD6 knockout (KO) cells, showing that E2F1 regulates distinct sets of gene in both conditions. Methylated E2F1 regulates genes that are responsible for prostate gland development, tumor suppression, and negative regulation of cell migration, while unmethylated E2F1 promotes cell proliferation and negative regulation of apoptosis. We show mechanistically that SETD6-mediated methylation at K117 disrupts the interaction of E2F1 with BRD4. Chromatin immunoprecipitation (ChIP) data confirm that SETD6 impacts the binding of E2F1 and BRD4 at the same target loci, establishing a novel mechanism by which SETD6 methylation of E2F1 modulates gene regulation programs in prostate cancer cells.

## Materials and methods

### BRD4 cloning, expression, and protein purification

The Glutathione-S-transferase (GST)-tagged expression construct of the truncated human BRD4 containing its BD1 and BD2 (amino acids 2-477, UniProt No O60885), its K99R mutant, and the GST-tagged expression construct of the truncated human BRD4 only containing BD1 (amino acids 2-220) were used for protein overexpression and purification. For protein overexpression, *Escherichia coli* BL21-CodonPlus cells (Novagen) were transformed with the GST-tagged constructs. The cells were grown in Luria–Bertani medium at 37°C until an OD_600_ of 0.6–0.8 was reached. The protein expression was induced with 1 mM isopropyl β-d-1-thiogalactopyranoside (Biovectra) overnight at 15°C. Cells were harvested by centrifugation at 4500 rpm, and the cell pellet was washed with STE buffer [10 mM Tris/HCl, pH 8.0, 1 mM ethylenediaminetetraacetic acid (EDTA), and 100 mM NaCl] and stored at −20°C. Cell pellets were resuspended in sonication buffer [50 mM Tris/HCl, pH 7.5, 150 mM NaCl, 1 mM Dithiothreitol (DTT), and 5% (w/v) glycerol] supplemented with protease inhibitor cocktail containing AEBSF–HCL (1 mM; Biosynth), Pepstatin (10 μM; Roth), Aprotinin (0.4 μM; Applichem), E‐64 (15.14 μM; Applichem), Leupeptin (22.3 μM; Alfa Aesar), and Bestatin (50 μM; Alfa Aesar) and lysed by sonication (14 cycles of 30 s at 30% power; Bandelin Sonopuls) at 4°C. Next, the sample was centrifuged at 18 000 rpm for 90 min at 4°C. Thereafter, the supernatant was loaded onto a Glutathione Sepharose 4B resin (GE Healthcare) column after pre‐equilibration of the beads with sonication buffer. After that, the beads were washed in two steps, first with sonication buffer and second with washing buffer [50 mM Tris/HCl, pH 8.0, 500 mM NaCl, 1 mM DTT, and 5% (w/v) glycerol], and the bound proteins were eluted with the washing buffer containing 40 mM glutathione. After elution, proteins were incubated in dialysis buffer I and II containing 20 mM Tris/HCl (pH 7.4), 100 mM KCl, 0.5 mM DTT, 10 or 60% (w/v) glycerol, respectively.

### Synthesis of peptide SPOT arrays

Peptide arrays were prepared using the SPOT synthesis technique using an Autospot Multipep peptide array synthesizer (CEM). Based on the Autospot Reference Handbook (CEM) each peptide spot contains ~9 nmol of peptide in a diameter of 2 mm. The successful synthesis of peptide arrays was validated by Bromophenol blue staining.

### BRD4 binding to peptide SPOT arrays

Peptide SPOT arrays containing 15 aa long E2F1 peptides with different modifications were blocked in 5% milk in 1X TBS-T (20 mM Tris/HCl pH 7.4 and 150 mM NaCl with 0.1% Tween 20 detergent) solution overnight. After blocking, the array was washed three times for 5 min with 1X TBS-T solution. Then, the array was preincubated in binding buffer containing 100 mM KCl, 1 mM DTT, 10% glycerol, 20 mM Hepes (pH 7.5), and 1 mM EDTA for 5 min on a shaker. Afterward, the array was incubated in binding buffer containing BRD4-WT (5 nM), BRD4-K117R (50 nM), BRD4-BD1 domain (5 nM) protein on a shaker for 1 h at room temperature. Next, the array was washed three times with 1X TBS-T for 5 min. After washing, the array was incubated with the primary GST antibody (Cytiva™, 27457701V 1:5000) for 1 h at room temperature. Before the array was incubated with the secondary antibody anti-goat HRP (Sigma, 1:5000) for 1 h at room temperature, it was again washed three times with 1X TBS-T for 5 min. After final washing, the signal was detected by chemiluminescence after the addition of Pierce™ ECL western-blotting substrate.

### Cell culture

The DU145 human prostate cancer cell line was kindly provided by Prof. Etta Livneh (Ben-Gurion University of the Negev, Israel). Cells were grown in Dulbecco’s modified Eagle’s medium (Sigma, D5671) media supplemented with 10% fetal bovine serum (FBS) (Gibco), 1% Penicillin-Streptomycin (Sigma, P0781), 2 mM L-glutamine (Sigma, G7513), and 1% nonessential amino acids (Sigma, M7145) at 37°C in a humidified incubator with 5% CO_2_. For the DU145 CRISPR/Cas9 generated SETD6 KO, single clones were confirmed by sequencing as described in previously [25].

### Transfection, immunoprecipitation, and western-blot analysis

The GFP-tagged BRD4 BD1-BD2 fragment and the Flag-tagged E2F1 (amino acids 2-437 of UniProt Q01094) were co-transfected into SETD6 WT and KO DU145 cells using polyethyleneimine (M.W. 25,000) (PEI, Alfa Aesar provided by Thermo Fisher Scientific) according to the instructions of the supplier. After 24 h of transfection, in some samples 2 µM of JQ1 (Thermo Fisher Scientific) was added and incubated with the cells for pharmacological inhibition of BRD4 binding to acetylated target proteins. After 72 h of transfection, the transfection efficiency and expression of the transgenes in DU145 cells were determined by flow cytometry (MACSQuant VYB, Miltenyi Biotec). The FlowJo software (Miltenyi Biotec) was used for data analysis. Next, cells were washed with 1× Phosphate Buffered Saline (PBS) buffer (Gibco) and harvested by centrifugation at 500 *g* for 5 min. After harvesting, the amount of proteins in whole cell lysate was determined by western-blot. For protein/protein interaction analysis, the GFP-fused proteins were purified from the cell extract using GFP-trap A beads (Chromotek) according to the manufacturer’s instructions. The eluted protein samples were incubated at 95°C for 5 min in sodium dodecyl sulfate (SDS)-gel loading buffer and subsequently separated on a 12% sodium dodecyl sulfate–polyacrylamide gel electrophoresis stained by Ponceau Red. For western-blot analysis, β-actin primary antibody (Santa Cruz, SC47778, 1:2500) was used as loading control. As secondary antibody, anti-mouse IgG-HRP (Cytiva, NXA931V,1:2500) was used. The Flag- and GFP-tagged proteins in the whole lysate and the eluted samples were determined by using an anti-Flag (Invitrogen, 1:2500) and anti-GFP antibody (Clontech, lot. 1404005, 1:2500) combined with the secondary antibodies anti-mouse IgG-HRP (Cytiva, NXA931V,1:2500) and anti-rabbit IgG-HRP (Cytiva, NA934, 1:2500), respectively.

Cell transfections were performed using polyethyleneimine (PEI) reagent (Polyscience Inc., 23966) or Mirus reagent TransIT-X2 according to the manufacturer’s protocol. For stable transfections in the DU145 cell line, retroviruses were produced by transfecting HEK293T cells with the indicated pWZL constructs (Empty, Flag-E2F1 WT, Flag-E2F1 K117R) with plasmids encoding VSV and gag-pol. DU145 cells were infected with the viral supernatants and cells with stable integration were selected with 200 μg/ml hygromycin B (TOKU-E).

### Chromatin immunoprecipitation and library generation for ChIP-seq

For ChIP-seq experiments, ~2 × 10^7^ cells were washed with 1× PBS buffer (Gibco) and harvested by centrifugation at 500 *g* for 5 min. Next, formaldehyde was added directly to the cell culture media to a final concentration of 2%. After 10 min incubation at room temperature, glycine was added to a concentration of 125 mM to quench the crosslinking reaction. After that, ChIP steps were conducted using the Magna ChIP™ HiSens Chromatin Immunoprecipitation Kit (Milipore, MAGNA0025) according to the manufacturer’s instructions. For each sample, 500 µl of Nuclei Isolation buffer and 500 µl of SCW (Sonication/ChIP/Wash) buffer were supplemented with protease inhibitors (200× Protease Inhibitor Cocktail III, Roche), by dissolving one tablet in water. The crosslinked pellet was resuspended in Nuclei Isolation Buffer, incubated on ice for 15 min with intermittent vortexing, and centrifuged at 800 *g* for 5 min at 4°C. The nuclear pellet was resuspended in 500 µl SCW Buffer with inhibitors. Chromatin was fragmented using the EpiShear™ Probe Sonicator (Active Motif) set to 20 ms pulses on and 30 ms off for 12 min at 40% amplitude, keeping the samples on ice at 4°C throughout the process. The lysates were centrifuged at 10 000 *g* for 10 min at 4°C, and the supernatant containing fragmented chromatin was collected for downstream analysis. For each ChIP, 10 μg Flag antibody (Invitrogen, F1804) and Mouse IgG antibody (Thermo Fisher, WE3290156) were pre-incubated with 20 µl Dynabeads coupled with Protein A (Life Technologies) in 0.5 ml PBS for 2 h at 4°C under gentle rotation. Chromatin from 10^7^ cells dissolved in 1 ml SCW (Sonication/ChIP/Wash) buffer was mixed with antibody beads complex and incubated overnight at 4°C under gentle rotation. Next day, the chromatin immunoprecipitates were washed twice with SCW buffer and then resuspended in 50 µl of ChIP Elution Buffer with Proteinase K. Afterward, the samples were incubated in a thermomixer at 65°C for 2 h and then at 95°C for 15 min. After incubation, the tubes were placed in a magnetic separator for 1 min, and 45 µl of the supernatant was transferred into a new microcentrifuge tube. To check successful enrichment, samples were characterized based on the size range of DNA by using LabChip DNA High Sensitivity Reagent kit (Perkin-Elmer, LabChip GXII Touch) and quantified by quantitative Polymerase Chain Reaction (qPCR), respectively. All library preparation steps were conducted according to manufacturer’s instructions of NEBNext^®^ Multiplex Oligos for Illumina^®^ (Index Primers Set 1) (NEB). The ChIP-seq library was send to Novogene (UK) for Next-Generation-Sequencing (Illumina Novaseq 6000 system, paired-end, 2 × 150 bp-mode). Two independent experiments were conducted for each condition.

In order to analyze how SETD6 influences the chromatin-binding of BRD4, and to compare it with E2F1 binding in SETD6 KO cells, BRD4 ChIP was performed using a BRD4-specific antibody (Abcam, ab243862) in SETD6 WT and KO DU145 cells. The experiments were conducted in accordance with the manufacturer’s protocol using the Magna ChIP™ HiSens Chromatin Immunoprecipitation Kit (Millipore, MAGNA0025).

### ChIP-qPCR

To validate the ChIP-seq experiments, ChIP-qPCR was performed for two known E2F1 binding regions (E2F1 and E2F8) [26–28] and one negative intergenic region close to the CALML5 gene [29]. Moreover, ChIP-qPCR was conducted to confirm BRD4 occupancy at selected genomic regions. The GAPDH transcription start site was used as a positive control, while an intergenic region was used as a negative control for BRD4-binding (taken from [30]). A Mouse IgG antibody (Thermo Fisher, WE3290156) pulldown was included as an additional negative control to assess immunoprecipitation specificity. As negative control of the qPCR, no template reactions were conducted. Primer sequences used for qPCR are listed in Supplementary Table S1. To normalize DNA input and evaluate PCR efficiency, a 1:5 serial dilution of input DNA was used.

For qPCR, 1:5 dilution series of the input DNA were prepared for normalization of the pulled-down DNA and evaluation of the PCR efficiency. All experiments were performed at least in three independent biological replicates. For each region of interest, a master mix was prepared including 7.5 µl of 2× ORA™ See qPCR Probe Mix (highQu), 0.4 µl forward primer, 0.4 µl reverse primer, and 5.7 µl ddH2O. For qPCR, 1 µl of each sample was used in triplicates for the input dilution series, IP, and nontemplate control. The following program was used: 95°C for 3 min, 35 cycles of 95°C for 3 s, 61°C for 20 s, 72°C for 4 s, and finally a 65°C–95°C ramp (0.5°C steps every 5 s) in a CFX Comet^TM^ Real Time System (BIORAD). Quality of the qPCR run was assessed by checking single melting curves to confirm the amplified product. Data were analyzed using the CFX Maestro software (BIORAD). Enrichment of samples were quantified by normalization of input.

### Chip-seq data processing

Sequencing data were received in FASTQ format and processed using the Galaxy Europe Server (https://usegalaxy.eu/). The read quality was analyzed by using FastQC. Adaptors were trimmed using Trimmomatic. HISAT2 was used for alignment to the GRCh38/hg38 reference genome. The resulting BAM file was used to generate a coverage BigWig file using 10 bp bin size and normalized by reads per kilobase per million mapped reads (RPKM). These processes were applied to two independent replicates as well as merged replicates 1 and 2 that were concatenated using MergeSamFiles. Reproducibility of the replicates and merged files (enriched Flag-E2F1 WT and input for negative control) were determined using multiBigWigSummary in 5 kb bins. The BigWig files were used to visualize peaks in Integrative Genomics Viewer (IGV) 2.13.1. To investigate differential binding sites of E2F1 in SETD6 WT or KO, E2F1 peaks were determined by using the MACS2 bdgpeakcall tool. The combined peaks were used to plot heatmaps and perform k-means clustering using the computeMatrix tool. Heatmaps were generated using the plotHeatmap tool. The EnhancerAtlas 2.0 database (http://www.enhanceratlas.org/) [31] was used to identify genes with regulatory elements overlapping with the genomic regions in Cluster 2 and Cluster 3 using the prostate cancer cell lines PC3 and LNCaP. The output of this annotation analysis was combined with RNA-seq data to identify correlations between differential binding of E2F1 and gene expression changes. EnhancerAtlas 2.0 was also used to retrieve the gene regulatory elements of candidate genes.

### Analysis of BRD4 signals in promoter and enhancer regions of genes

Promoter and enhancer regions of gene in Cluster 2 and Cluster 3 were extracted from EnhancerAtlas 2.0 database. To compare the BRD4 signal distribution across these regions, the computeMatrix tool (Galaxy Version 3.5.4+galaxy0) was used to generate profiles for the different gene sets. The numerical output was used for the calculation of the highest BRD4 signal among the elements associated with each gene. The plotProfile tool (Galaxy Version 3.5.4+galaxy0) was used to create heatmaps to show the average signal.

### Library generation for RNA-seq

For RNA-seq experiments, total RNA was isolated from approximately 1 × 10^6^ cells by using the RNeasy^®^ Mini Kit (Qiagen). The extracted RNA was quantified by NanoDrop1000 and reverse-transcribed into complementary DNA (cDNA) to generate RNA-seq libraries. Library generation was performed with the NEBNext^®^ Ultra™ II Directional RNA Library Prep Kit for Illumina^®^ (NEB), along with NEBNext^®^ Multiplex Oligos for Illumina^®^ (Index Primers Set 1) (NEB). All library preparation steps were conducted according to the manufacturer’s instructions. The quality and concentration of RNA-seq libraries were determined by Labchip^®^ GX Touch (Perkin Elmer) for fragment size distribution and concentration analysis. Finally, each RNA library was sequenced by Novogene (UK) using an Illumina Novaseq 6000 system in paired-end 2 × 150 bp-mode. Three independent RNA-seq experiments were processed for each condition.

### RNA-seq data processing

Sequencing data were received in FASTQ format and processed by using Galaxy Europe Server (https://usegalaxy.eu/). The read quality was analyzed by using FastQC. Adaptors were trimmed using the Trimmomatic tool. HISAT2 was used for alignment to the GRCh38/hg38 reference genome. Reads that overlapped with features in the corresponding GFF file (Homo Sapiens-GRCh38.87.gtf) were counted. DESeq2 [32] was used to determine significant differentially expressed genes (DEGs) in E2F1 WT/K117R and SETD6 WT/KO cells in default settings. The DEGs were visualized in a heatmap created using the Heatmapper.ca web tool [33]. Gene Ontology (GO) enrichment analysis was performed using data sets combined from Chip-seq and RNA-seq focusing on genes with strong binding side of E2F1 and upregulation in a SETD6 WT or KO context. GO analysis was performed with ShinyGO 0.8 (https://bioinformatics.sdstate.edu/go80/) [34] and Enrichr (https://maayanlab.cloud/Enrichr/) [35].

### RNA extraction and reverse-transcription qPCR (RT-qPCR)

Total RNA was extracted using the Monarch Total RNA Miniprep Kit (NEB). The extracted RNA (1.5 µg) was reverse-transcribed to cDNA using a reaction mixture containing 2 µl 10× RT buffer, 40 mM dNTPs, 2 µl diluted Random Hexamers (1:10), 1 µl RNase inhibitor (1:10), 25 mM MgCl₂, and 1 µl MultiScribe™ Reverse Transcriptase (Thermo) in a final volume of 20 µl. Reverse transcription was performed at 25°C for 10 min, followed by 37°C for 120 min and 85°C for 5 min. Each qPCR reaction contained 7.5 µl of 2× ORA™ See qPCR Probe Mix (highQu), 0.4 µl of forward primer (10 µM), 0.4 µl of reverse primer (10 µM), and 5.7 µl of ddH₂O. Reactions were prepared in triplicate, using 1 µl of cDNA per well. qPCR was carried out on a CFX Comet™ Real-Time System (BIO-RAD) under the following cycling conditions: initial denaturation at 95°C for 3 min, followed by 39 cycles of 95°C for 3 s, 61°C for 20 s, and 72°C for 4 s. A melting curve was generated from 65°C to 95°C with 0.5°C increments every 5 s. Data were analyzed using the CFX Maestro software (BIO-RAD), and gene expression levels were normalized to GAPDH. Primer sequences used in this study are listed in Supplementary Table S2.

### Colony formation assay

For colony formation assay, DU145 cells were plated on a 12-well plate at 1000 cell/well density. After 14–18 days the cells were fixed and stained by crystal violet staining solution (0.5% crystal violet in 20% methanol). Crystal violet staining was solubilized in 2% SDS and quantified at 550 nm using Tecan Infinite M200 plate reader.

### Cell migration assay

DU145 cells were serum starved (0.5% FBS) overnight, then 3.5 × 10^5^ cell/well were plated on a 48-well plate. Next, a scratch wound was produced by dragging a 200-µl pipette tip across the layer cells. The migrated distance of the cells toward the gap was monitored by a Lionheart™ FX Automated Microscope (4×).

### Apoptosis assay

Different DU145 cell lines were either treated or not treated with Doxorubicin (DOX) (2.5 µM/5 µM) for 5 h followed by media change and overnight cultivation before harvesting. Media was collected from treated or untreated cells and followed by cell harvesting using trypsin and cell staining with Annexin V-FITC (1:200) and PI (1:400) in 200 µl Annexin V binding buffer using a Biolegend Apoptosis kit. Samples were analyzed by flow cytometry (Guava^®^ easyCyte flow cytometer). Apoptosis was defined as the percentage of cells positive for both PI and Annexin V. All experiments were conducted in two biological repeats, each of them including three technical replicates.

### Proximity ligation assay

Cells were cultivated on coated coverslips, washed with PBS, and fixed in cold 4% paraformaldehyde at RT for 15 min. Cell permeabilization was performed using 0.5% Triton X-100 in PBS for 10 min at ambient temperature. PLA Duolink assays were performed according to the manufacturer’s instructions (Sigma) using antibodies against BRD4 (Bethyl, A700-004), E2F1 (SantaCruz, SC-251) and Flag (Sigma, F1804) overnight at 4°C. Images were acquired by confocal Spinning disk microscopy with a 63× objective. Each frame represents a maximum intensity projection for Z-stacks captured, and 4–8 frames were captured for each sample. The proximity ligation assay (PLA) units were calculated per cell as the ratio between the number of dots within the nucleus and the nucleus area (stained with DAPI, using Duolink mounting media). Each nucleus was represented as a point in the quantification graph. Where indicated, SAHA (Sigma, SML0061) was added to the cells prior to the cell fixation.

For the automated counting of PLA emitters per nucleus in batch mode, an ImageJ (Fiji) macro was used [36]. In each image, the nuclei were first segmented by applying a Gaussian blur and using standard thresholding algorithms, such as Otsu. The segmented nuclei were then converted into ImageJ ROIs using the MorpholibJ [37] and PTBIOP (https://www.epfl.ch/research/facilities/ptbiop/image-processing/) plugins. For each ROI (nucleus), the number of PLA emitters was determined by identifying local maxima following blob detection via the difference of Gaussian algorithm.

### Statistics and reproducibility

*P*-values were determined by *t*-test with Excel or GraphPad (Version 8) using parameters as indicated. The number of experimental repeats is indicated in each case. Statistical analysis of the apoptosis and wound healing assays was performed using two-way ANOVA with GraphPad.

## Results

### SETD6 methylation regulates chromatin binding of E2F1

**Figure 1.**
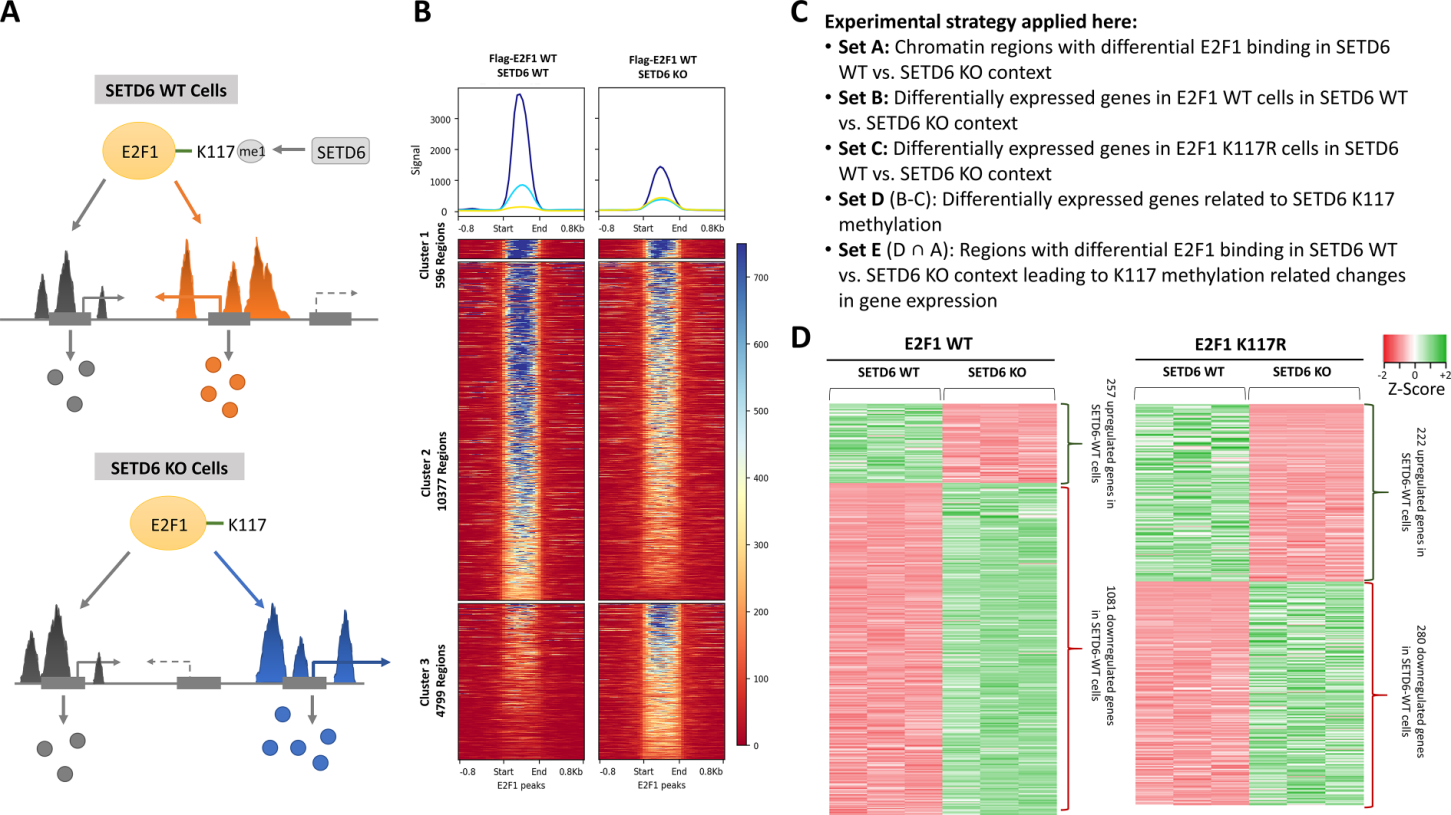
SETD6-mediated methylation of E2F1 at K117 regulates the binding site of E2F1 and genes expression. (**A**) The E2F1 transcription factor is methylated by SETD6 at K117. This research investigated whether methylation of E2F1 K117 by SETD6 has a role in regulating global gene expression programs. (**B**) Heatmap of RPKM-normalized E2F1 ChIP-seq signals at combined E2F1 peak regions (±0.8 kb) showing differential binding of E2F1 in DU145 prostate cancer cells stably expressing Flag-E2F1 in SETD6 WT or KO context. Separation into three clusters by *k*-means clustering revealed cluster 1 containing regions bound similarly by E2F1 in SETD6 WT and KO cells (596 regions), cluster 2 containing 10377 regions with stronger binding in the SETD6 WT cells, and cluster 3 containing 4799 regions with stronger binding in SETD6 KO cells. Data were merged from two experimental repeats, see Supplementary Fig. S6. (**C**) Summary of the experimental strategy to identify candidate genes that are differentially bound and differentially expressed in a K117 methylation dependent manner. (**D**) RNA-seq experiments conducted in DU145 SETD6 WT and KO cells expressing Flag-E2F1 or E2F1 K117R. Differential gene expression was analyzed by Heatmapper (http://heatmapper.ca/). The heatmaps display significant differential expressed genes (log_2_-fold change >1.5 and *P*-value <0.05) in SETD6-WT and KO cells either expressing E2F1 WT or E2F1 K117R. Green color represents up-regulated genes and red color represents down-regulated genes. Three replicates were analyzed for each condition. A scatter plot showing the results of a principal component analysis (PCA) of transformed count data from the RNA-seq replicates using DESeq2 is provided in Supplementary Fig. S7. The source data to Fig. 1B and D are provided in Supplementary Data S1.

E2F1 is a transcription factor with important roles in the regulation of oncogenic signalling pathways, including cell cycle progression, apoptosis, and DNA repair, which influences cancer-related phenotypes in various malignancies including prostate cancer [38–42]. One primary mechanism affecting E2F1 involves the dysregulation of the RB pathway where RB depletion leads to aberrant E2F1 chromatin interaction [43, 44]. However, the post-translational regulation of E2F1 including its acetylation and methylation is another important regulatory process. In our previous study, we described that SETD6 methylates E2F1 at K117 *in vitro* and in prostate cancer cells. Additionally, monomethylated E2F1 was shown to bind to the SETD6 promoter and by this increase SETD6 expression creating a positive feedback loop [25]. Based on these data, we were interested to find out whether methylation of E2F1 K117 by SETD6 has a more general role in the regulation of gene expression programs in prostate cancer cells (Fig. 1A). To test this hypothesis, we used DU145 prostate cancer cell lines stably expressing Flag-E2F1 WT or Flag-E2F1 K117R mutant in SETD6 WT and SETD6 KO context that have been described in our previous study [25] and were characterized with respect to the expression of SETD6 and the different forms of E2F1 (Supplementary Fig. S1). The four different cell lines were used to investigate the biological functions of E2F1 and SETD6 and their potential interactors at the molecular level by using multiple genomic approaches including ChIP-seq and RNA-seq (Fig. 1). Comparison of the data obtained in these cell lines provides detailed information of how E2F1 behaves in the absence of SETD6 or after its monomethylation by SETD6.

First, we conducted ChIP-qPCR experiments to analyze E2F1 genome binding in SETD6 WT and KO DU145 cells. Initially, E2F1 binding was analyzed at three different loci by qPCR, two positive ones (E2F1 and E2F8 promoter regions) and an intergenic region close to the CALML5 gene as negative control. As expected, high enrichment of E2F1 was observed at the E2F1 and E2F8 regions in ChIP samples, but not in IgG controls, while no enrichment was determined at the negative control region (Supplementary Fig. S2 and  Supplementary Table S1). Next, E2F1 ChIP-seq data were obtained from Flag-E2F1 in SETD6 WT and KO context in two replicates, together with input samples as negative control. The two experimental repeats of SETD6 WT and SETD6 KO, respectively, correlated strongly, but they were different from each other and also from input (Supplementary Fig. S3A and B). Based on this, we conclude that SETD6 dependent differential chromatin binding of E2F1 occurs at many genomic loci, exemplary browser views are shown in Supplementary Fig. S4A.

In the individual replicates of the E2F1 ChIP-seq experiments in SETD6 WT and KO cells, peaks were identified using MACS2 and manually validated (Supplementary Fig. S4B). The peaks were then merged resulting in 12929 peaks for Flag-E2F1 in SETD6 WT. Conversely, the Flag-E2F1 in SETD6 KO yielded 13780 combined peaks, both sets combined contained 15778 peaks indicating reproducible peak calling. To assess the reproducibility and biological relevance of the E2F1 binding sites identified in SETD6 WT DU145 cells, we compared our findings with published E2F1-ChIP-seq datasets derived from other prostate cancer cells (dataset SRR951091 taken from GSE49832 [45] and dataset SRR24060765 taken from GSE228895 [46]). A heatmap was generated to visualize RPKM-normalized E2F1 ChIP-seq signals at E2F1 peaks (±0.8 kb) in SETD6 WT DU145 cells alongside with the published datasets that revealed high correlation (Supplementary Fig. S5).

Next, we aimed to detect regions with differential binding of E2F1 in SETD6 WT and KO cells. For this, a heatmap was generated by RPKM-normalized ChIP-seq signals in all samples using the merged E2F1 peaks detected in SETD6 WT and KO cells (Supplementary Fig. S6A). To identify regions with higher E2F1 signals in SETD6 WT or KO cells, *k*-means clustering was conducted for three clusters (Fig. 1B and Supplementary Fig. S6B). Using this approach, we detected similar E2F1 binding in both WT and SETD6 KO cells in cluster 1 (596 regions). Cluster 2 contains 10377 regions that were more strongly bound in SETD6 WT conditions suggesting that they are bound by methylated E2F1 or E2F1 binding is indirectly stimulated by other SETD6-dependent factors. In contrast, stronger E2F1 binding in SETD6 KO cells was observed at 4799 regions in cluster 3 (Fig. 1B). This indicates that these sites may preferentially bind unmethylated E2F1 or that E2F1 binding at these loci is indirectly influenced by other SETD6-dependent factors. In summary, ChIP-seq led us to the identification of potential regions that were differentially bound by E2F1 depending on SETD6 activity. The EnhancerAtlas 2.0 tool (http://www.enhanceratlas.org/) [31] was used to associate the regions preferentially bound by E2F1 in SETD6 WT and KO context with gene regulatory elements and identify the corresponding genes. This analysis retrieved 8765 and 4158 genes in both cases that were then used in the next step of the analysis aiming to explore how SETD6-dependent E2F1 binding influences gene expression.

The experimental strategy applied in this work to identify candidate genes that are differentially bound and differentially expressed in an E2F1-K117 methylation dependent manner is summarized in Fig. 1C. As described so far, E2F1 ChIP-seq studies in SETD6 WT and KO context allowed us to define a set of gene control elements that were differentially bound (Set A). Next, we were interested to identify the potential biological effects of the SETD6 methylation dependent differential E2F1 binding. As gene regulation is the most straightforward role of E2F1, in the next chapter we investigated gene expression in the presence of overexpressed E2F1 in SETD6 WT and KO cells and identified DEGs (Set B). However, SETD6 KO is known to have many potential effects on gene expression that are unrelated to E2F1 K117 methylation. Therefore, we also determined gene expression in the presence of the overexpressed E2f1 K117R mutant, which cannot be methylated by SETD6 and determined SETD6 WT versus KO DEGs in this context (Set C). To focus on DEGs related to E2F1 K117 methylation, we next created Set D of genes that are differentially expressed in E2F1 context but not in E2F1 K117R context (Set B–Set C). Finally, we intersected these genes with genes showing differential binding of E2F1 in SETD6 WT versus KO context generating Set E as the filtered candidate genes where differential binding of E2F1 in SETD6 WT versus KO context is correlated with differential gene expression in the E2F1 WT context but not with the E2F1 K117R mutant.

### SETD6-dependent E2F1 transcriptional regulation affects many cellular processes in prostate cancer cells

To determine differential gene expression by E2F1, RNA-seq was performed in triplicate in the DU145 cells stably overexpressing Flag-E2F1 WT in SETD6 WT and SETD6 KO background. To identify genes with expression changes independent of E2F1 methylation, gene expression was also analyzed in SETD6 WT and SETD6 KO cells expressing the E2F1 K117R mutant, which cannot be methylated by SETD6 (Fig. 1D). While these cell lines all contain endogenous E2F1, the higher expression levels of the Flag-E2F1 WT and K117R mutant (Supplementary Fig. S1) ensure that the overexpressed E2F1 proteins have a dominating effect on our data. The RNA-seq data showed strong correlation among replicates clustering together in PCA and in the sample distance matrix (Supplementary Fig. S7). The final analysis of significant DEGs (log_2_-fold change >1.5 and *P*-value <.05) showed 1081 downregulated and 257 upregulated genes in E2F1 WT expressing SETD6 WT cells (Fig. 1D). Additionally, there are 222 upregulated and 280 downregulated genes in E2F1 K117R expressing SETD6 WT cells.

**Figure 2.**
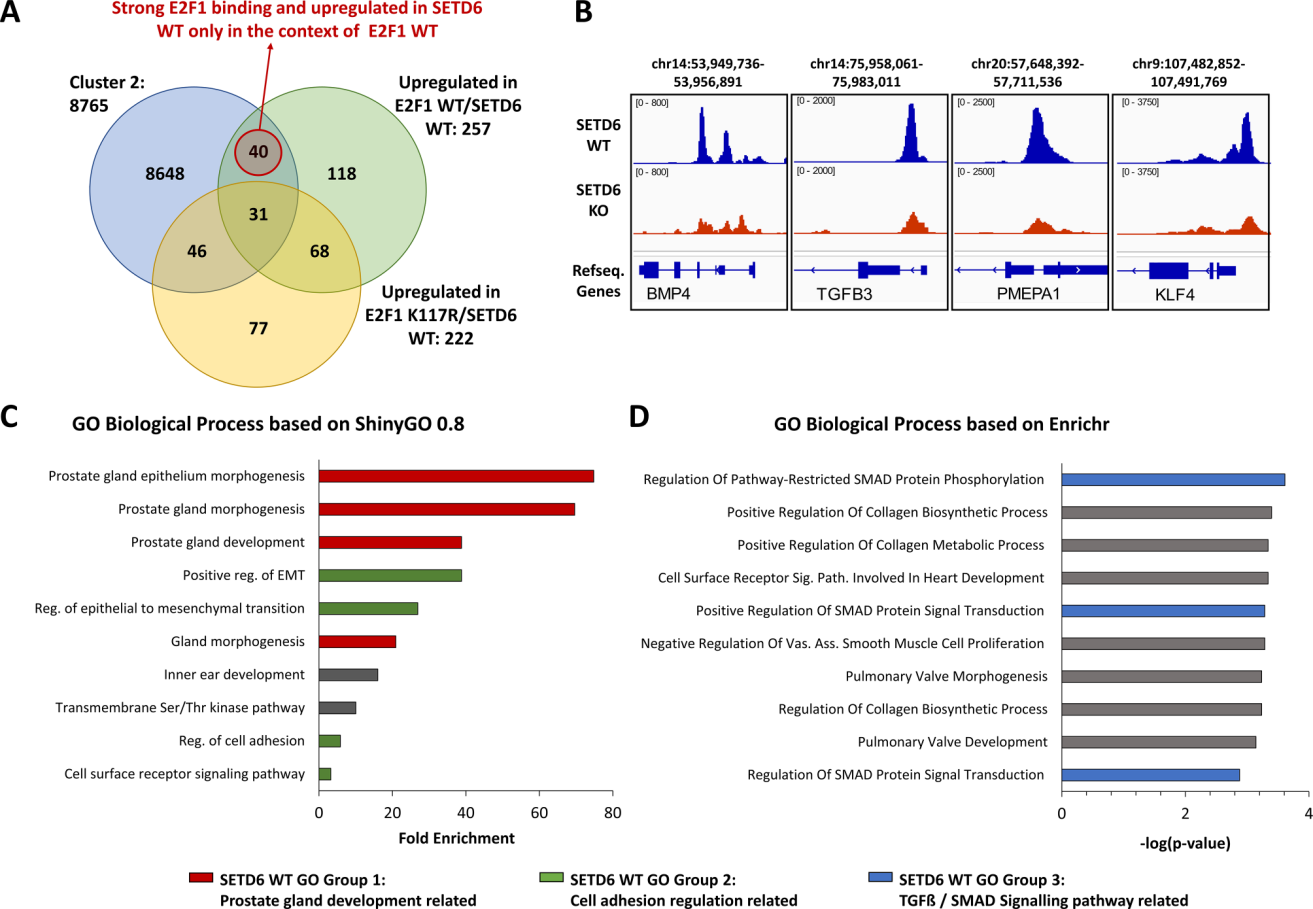
Integration of ChIP-Seq and RNA-Seq datasets identifies potential K117me dependent targets of E2F1 and corresponding cellular processes in prostate cancer cells. (**A**) Venn diagram showing the overlap genes of stronger ChIP-seq peaks in SETD6 WT and RNA-seq results. Fig. 1B cluster 2 related genes (8765) showing strong E2F1 binding in SETD6-WT (blue), 257 upregulated genes in SETD6 WT cells overexpressing Flag-E2F1 WT (green), and 222 upregulated genes in SETD6 WT cells overexpressing Flag-E2F1 K117R (yellow) have been identified. Finally, 40 genes show strong E2F1 binding and upregulation with E2F1 WT but not with E2F1 K117R. The Venn diagram is not drawn to scale. (**B**) Example browser views showing E2F1 binding to genes upregulation in E2F1 WT only in the context of SETD6 WT. ChIP-seq tracks of E2F1 in SETD6 WT (blue) and KO (red) cells are displayed. (**C**) GO biological pathway enrichment analysis on gene sets using the 40 genes identified in panel (A) conducted by ShinyGO 0.8. (**D**) GO biological pathway enrichment analysis on gene sets using the 40 genes identified in panel (A) conducted by Enrichr. The genes cluster in three GO groups related to roles in prostate gland morphogenesis and development (red), cell adhesion (green), and TGF-β-SMAD signaling (blue). For exemplary genes and gene functions see Table 1.

The combination of the ChIP-seq and RNA-seq data provides complementary insights into gene regulatory networks leading to deeper insights into biological effects. Specifically, we identified loci with stronger E2F1 binding site on genes either in SETD6 WT or SETD6 KO cells in Cluster 2 and Cluster 3, providing insights into the role of SETD6 in regulating E2F1-mediated transcription. To explore this, we mapped DEGs and genes with differential E2F1 binding to assess the impact of E2F1 methylation on gene expression. Comparison of the 8765 genes with enhanced E2F1 binding in SETD6 WT cells and 257 genes upregulated E2F1 WT SETD6 WT revealed 71 genes in the overlap. From those, 31 genes were also upregulated by E2F1 K117R that were excluded as their regulation was not dependent on K117 methylation. Finally, 40 genes were found to be both strongly bound by E2F1 and specifically upregulated in SETD6 WT cells in the context of E2F1 WT (Fig. 2A and B, and Supplementary Table S3 for the list of the genes in this group). GO gene set enrichment analysis (GSEA) performed with ShinyGO 0.8 [34] and Enrichr [35] revealed that these 40 genes are involved in prostate gland development, morphogenesis, regulation of cell adhesion, and TGFB-SMAD signaling pathway (Fig. 2C and D). Of note, the gene set includes several genes shown to have pro-apoptotic function and negative effects on cell proliferation in prostate cells (Fig. 2B and Table 1). Among these, the BMP4 (Bone morphogenetic protein 4) and TGFB3 (Transforming growth factor beta 3) genes are in all three GO groups. These genes are crucial regulators of prostate gland development and morphogenesis and have pro-apoptotic effects in prostate cancer cells [47, 48].

**Table 1.** Roles of SETD6-regulated E2F1 target genes in prostate and prostate cancer cells

	Gene	Gene name	Role of genes	Ref.
Forty genes in SETD6 WT group	KLF4	KLF transcription factor 4	Pro-apoptotic, suppresses migration and invasion in prostate cancer	[49–51]
	RNF217	Ring Finger Protein 217	Tumor suppressor gene in prostate cancer	[52]
	BMP4	Bone morphogenetic protein 4	Pro-apoptotic in prostate cancer (in WT GO groups 1, 2, and 3)	[47]
	TGFB3	Transforming growth factor beta 3	Pro- apoptotic, anti-proliferative in prostate cancer (in WT GO groups 1, 2, 3)	[48]
	CDH1	E-cadherin	Reduces cell migration and invasion in prostate cancer (in WT GO group 2)	[53]
	PMEPA1	Prostate transmembrane protein androgen induced 1	Tumor suppressor depending on isoforms in cancer (in WT GO groups 2 and 3)	[54]
Two hundred ten genes in SETD6 KO group	PTK6	Protein tyrosine kinase 6	Anti-apoptotic, promotes cell proliferation and migration in prostate cancer	[55, 56]
	PHB2	Prohibitin 2	Promotes prostate cancer progression and cell migration	[57]
	COX10	Cytochrome C oxidase assembly factor COX10	Deletion of gene prevents prostate cancer progression (in KO GO group 1)	[58]
	DHX37	DEAH-box helicase 37	Highly expressed in prostate cancer (in KO GO group 1)	[59]
	DCUN1D5	Domain containing E3 ubiquitin protein ligase 5	Promotes cell growth and migration in prostate cancer (in KO GO group 2)	[60]
	UBE2S	Ubiquitin conjugating enzyme E2S	Promotes prostate cancer progression (in KO GO group 2)	[61]
	CDC20	Cell division cycle 20	Induces cell proliferation and migration (in KO GO group 2)	[62]
	TOPORS	Topoisomerase I binding, arginine/serine-rich	Negative regulator of NKX3.1 TSG, induces prostate cancer progression (in KO GO group 2)	[63]
	SPHK2	Sphingosine kinase 2	Promotes prostate cancer progression (in KO GO group 3)	[64]
	ALDH3B1	Aldehyde dehydrogenase 3 family member B1	Against oxidative stress (in KO GO group 3)	[65]
	PRKD2	Protein kinase D2	Induces colony formation and metastasis in prostate cancer (in KO GO group 3)	[66]

**Figure 3.**
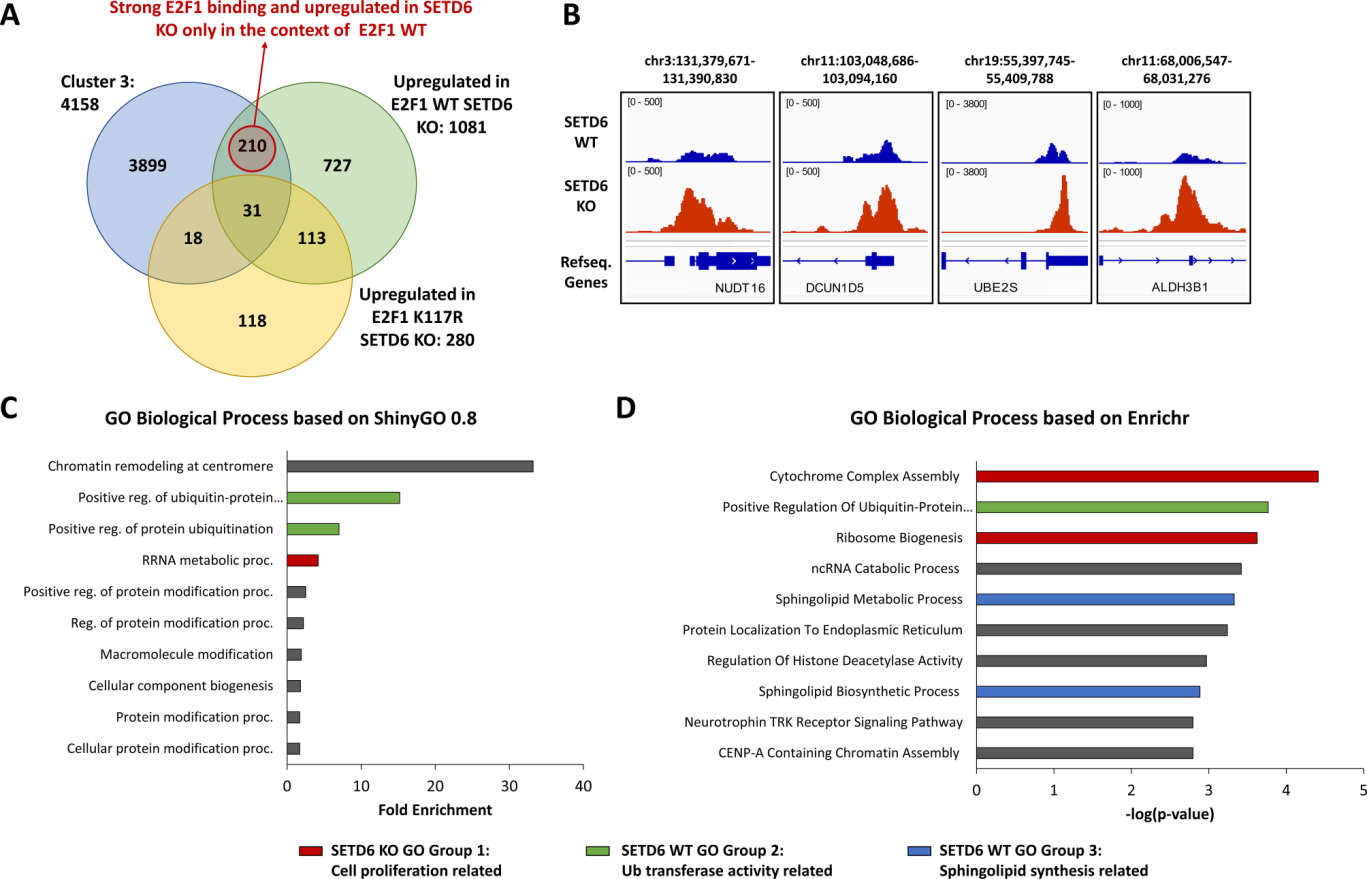
Integration of ChIP-Seq and RNA-Seq datasets identifies direct targets of E2F1 in SETD6 KO cells and corresponding cellular processes. (**A**) Venn diagram showing the overlap genes with stronger ChIP-seq binding in SETD6 KO and RNA-seq results. Fig. 1B cluster 3 related genes (4158) showing strong E2F1 binding in SETD6-KO (blue), 1081 upregulated genes in SETD6 KO cells overexpressing Flag-E2F1WT (green), and 280 upregulated genes in SETD6 KO cells overexpressing Flag-E2F1 K117R (yellow) have been identified. Finally, 210 genes show strong E2F1 binding and upregulation with E2F1 WT but not with E2F1 K117R. The Venn diagram is not drawn to scale. (**B**) Example browser views showing genes with strong E2F1 binding and upregulation in E2F1 WT only in the context of SETD6 KO. ChIP-seq tracks of E2F1 in SETD6 WT (blue) and KO (red) cells are displayed. (**C**) GO biological pathway enrichment analysis on gene sets using the 210 genes identified in panel (A) conducted by ShinyGO 0.8. (**D**) GO biological pathway enrichment analysis on gene sets using the 210 genes identified in panel A conducted by Enrichr. The genes cluster in three GO groups related to cell proliferation (red), ubiquitin transferase activity (green), and sphingolipid biosynthesis processes (blue). For exemplary genes and gene functions see Table 1.

In SETD6 KO cells, 241 genes showed stronger E2F1 binding and upregulation of gene expression. After removing 31 genes that were also upregulated with the E2F1 K117R mutant, 210 genes were found to be strongly bound by E2F1 and upregulated in SETD6 KO only in the context of E2F1 WT (Fig. 3A and B, and Supplementary Table S4 for the list of genes in this group). GO analysis (Fig. 3C and D) identified several genes involved in prostate cancer regulation, progression, and metastasis (Fig. 3B and Table 1). SETD6 KO GO Group 1 is related to cell proliferation and includes DHX37 and COX10, which both are associated with prostate cancer (Table 1). SETD6 KO GO Group 2 contains genes promoting prostate tumor growth and migration via regulation of ubiquitin transferases, including DCUN1D5, UBE2S, and CDC20 (Table 1). GO group 3 contains genes with roles in sphingolipid metabolism including SPHK2, which has been shown to promote prostate cancer [64]. These results suggest that SETD6 loss influences multiple oncogenic pathways. The discovery of these distinct sets of E2F1 target genes specifically upregulated in the presence or absence of SETD6 illustrates the role of E2F1 K117 methylation by SETD6 in modulating E2F1-driven transcription of distinct sets of target genes.

### Cellular assays in SETD6 WT and KO DU145 cells

**Figure 4.**
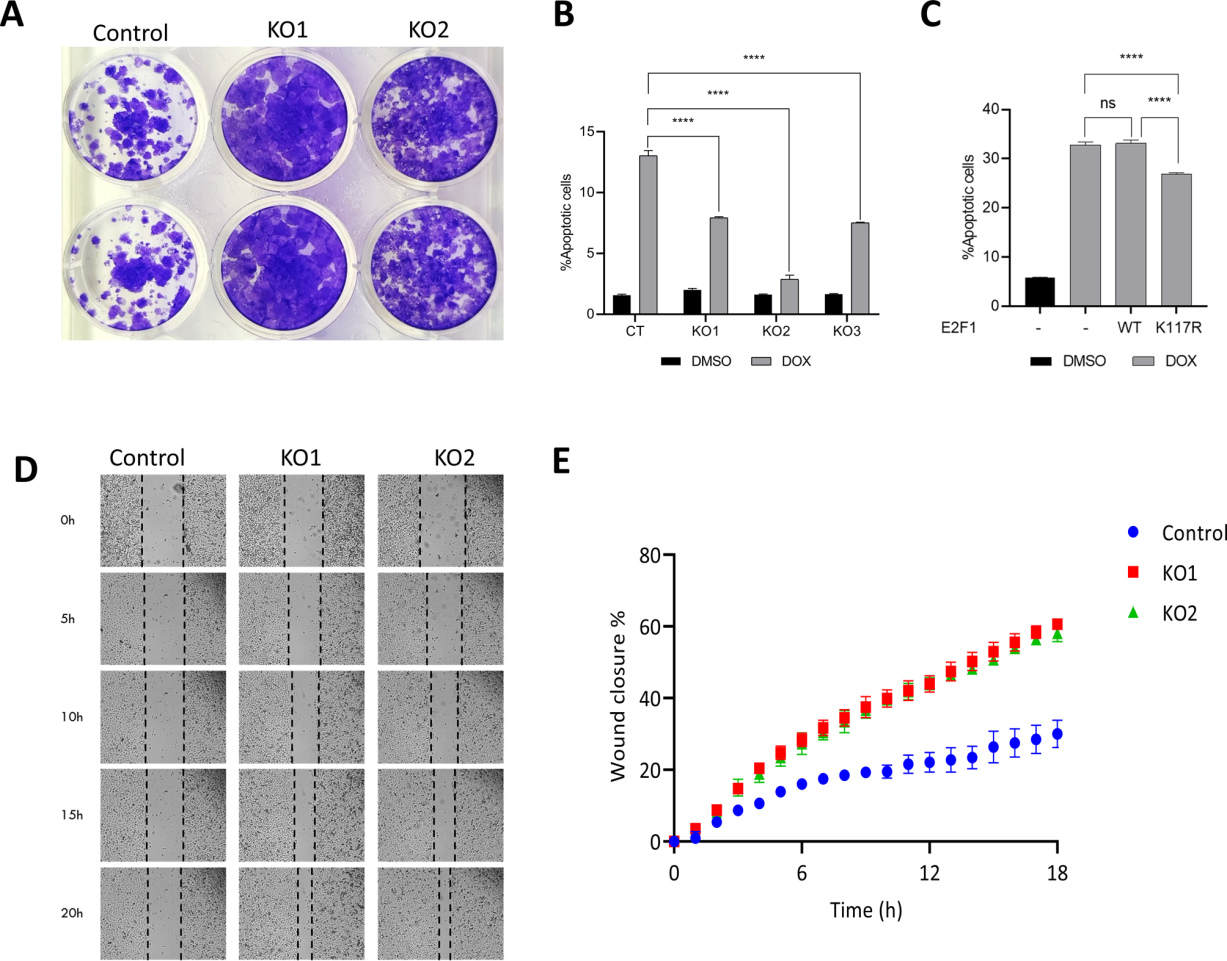
Cellular processes in prostate cancer cells regulated by SETD6 activity. (**A**) Exemplary images of colony formation assays performed in DU145 SETD6 WT (Control) and KO cells. Images were captured 16 days after cell plating. (**B**) Apoptotic response in DU145 WT (Control) and three independent KO cell lines indicated as fraction of apoptotic cells. Black bars: DMSO treated cells used as negative control. Gray bars: 2.5 µM DOX-treated cells. (**C**) Apoptotic response in DU145 WT either empty (−) or with stable expression of WT Flag-E2F1 (WT) or its K117R mutant (K117R) indicated as fraction of apoptotic cells. Black bars: DMSO treated cells used as negative control. Gray bars: 2.5 µM DOX-treated cells. In panels (B) and (C), statistical analysis was performed for duplicates using two-way ANOVA. ****P < .0001. Error bars show the deviation of three technical replicates. For biological replicates see Supplementary Fig. S8C and D. (**D**) Cell migration was detected by wound healing tests in SETD6 WT cells and two SETD6 KO clones. Representative images are shown captured 0, 5, 10, 15, and 20 h after scratch producing with black dashed lines indicating wound borders. (**E**) Quantitative analysis of the data shown in panel (E). Blue dots: SETD6 WT (Control) cells. Green and red dots: two SETD6 CRISPR KO cells derived from two independent gRNAs clones. Exemplary primary cytometry data for panels (B) and (C) are shown in Supplementary Fig. S8.

To investigate whether E2F1 regulates cellular processes in a SETD6-dependent manner in prostate cancer cells, colony formation was assayed in SETD6 WT and KO cells, which represent the methylated and unmethylated states of E2F1. As shown in Fig. 4A, increased colony formation was observed with SETD6 KO cells. In addition, an apoptosis assay was performed in SETD6 WT and KO cells treated by Doxorubicin (DOX). The anti-tumor drug DOX is used in clinical treatment of different types of cancer to induce apoptosis via the formation of reactive oxygen species [67–69]. As shown in Fig. 4B, a significant decrease in DOX-mediated apoptosis was detected in SETD6 KO cells. Hence, the depletion of SETD6 led to reduced apoptosis and enhanced colony formation. Apoptosis assays with DU145 cells stably expressing E2F1 WT or K117R mutant indicate that the K117R mutant expressing cells showed reduced apoptosis when compared to E2F1 WT expressing cells (Fig. 4C). These data show that SETD6 KO and K117R both lead to a reduction in apoptosis. This result suggests that the elevated apoptosis in the presence of SETD6 is at least in part due to K117 methylation of E2F1, although we cannot exclude effects mediated by other SETD6 substrates and pathways. Afterward, the role of SETD6-depended mechanisms in normal prostate development was investigated in wound healing assays showing a significant increase in cell migration in two SETD6 CRISPR KO cells derived from two independent clones (Fig. 4D and E). We conclude that SETD6 mediated E2F1 methylation induces the expression of genes that are pro-apoptotic, and involved in negative regulation of colony formation and cell migration.

### Monomethylation of K117 prevents E2F1 binding to BRD4

Post-translational modifications such as phosphorylation, ubiquitination, acetylation, and methylation are known to regulate protein–protein interactions [70]. The region surrounding the K117 site of E2F1 is a PTM hotspot with arginine methylation (R109, R111, and R113), lysine acetylation (K117, K120, and K125), and S121 phosphorylation listed in PhosphositePlus [71] suggesting that there could be an intensive crosstalk between the PTMs in this region and K117me. To further explore this, we focused on the fact that K117me antagonizes K117ac. It was previously shown that the acetylation of E2F1 at K117 and K120 enables its binding to BRD4 [24]. In agreement with this, active E2F1/BRD4 transcriptional programs have been identified in prostate cancer cells [72] and cellular data have documented direct effects of BET proteins on E2F1 transcriptional activities [43].

**Figure 5.**
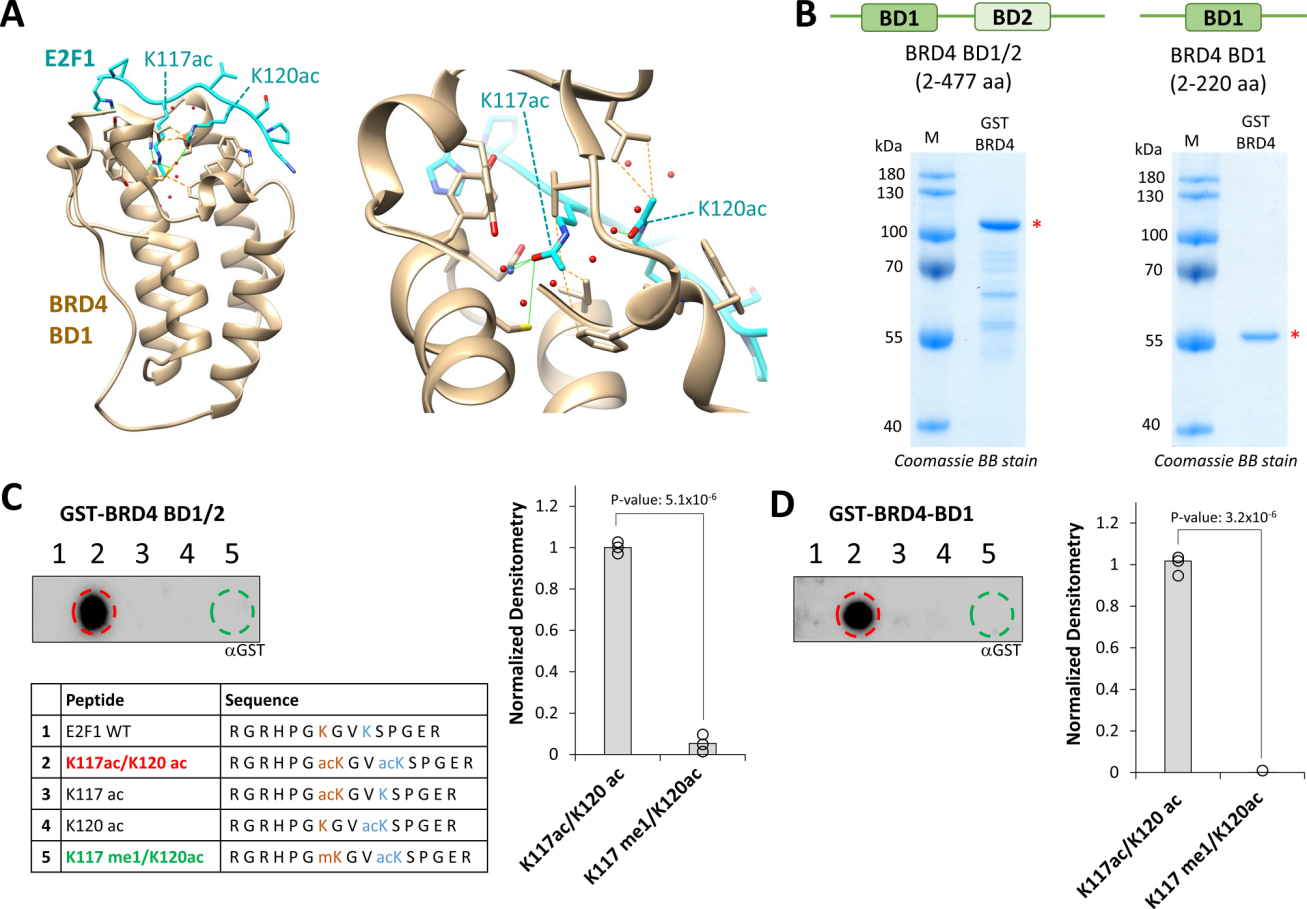
E2F1-BRD4 binding is lost with K117 monomethylated E2F1 *in vitro*. (**A**) Crystal structure of the human BRD4 BD1 (sandy-brown) in complex with an acetylated K117ac/K120ac E2F1 peptide (blue) (PDB 6ULS) [24] showing the key interaction of K117ac with BD1. (**B**) Coomassie BB stained 12% SDS gel of the purified GST tagged truncated BRD4 (2-477 aa) protein including BD1 and BD2 (BD1/2) as well as the purified BD1 domain (2-220 aa). The GST-tagged BRD4 proteins are marked with asterisks. (**C**) Binding of the GST-BRD4 BD1/2 to modified E2F1 peptides. 15 aa long E2F1 peptides with different combinations of unmodified, acetylated, and methylated K117 and K120 were synthesized on peptide SPOT arrays. The sequence of each peptide is listed in the table. Peptide arrays were incubated with 5 nM GST-BRD4 BD1/2 and binding was detected using a GST-specific antibody. The bar diagram shows the binding of E2F1-BRD4 to K117ac/K120ac and K117me/K120ac observed in three independent experiments. The bars represent the averages. The *P-*value was determined by two flanked *t*‐test with equal variance. (**D**) Same as in panel (C), but GST-BRD4 BD1 was used. Additional data are provdied in Supplementary Fig. S9.

A structural analysis of the interaction of E2F1 acetylated at K117 and K120 with the first BD of BRD4 (BD1) [24] suggests that K117ac acts as primary contact point of BD1 while K120ac provides a secondary contact (Fig. 5A). Therefore, we hypothesized that E2F1 K117 methylation may disrupt BRD4 binding to E2F1, because it prevents K117ac, thereby establishing a direct regulation of the BRD4–E2F1 interaction by SETD6 activity.

Therefore, we conducted a peptide array binding experiment using the E2F1 peptide (111–125) with different modifications. For this, truncated GST-tagged human BRD4 proteins, containing either the BD1 domain or BD1 and BD2, were recombinantly expressed in *E. coli* and purified by affinity chromatography with high yield and purity (Fig. 5B). As shown in Fig. 5C and Supplementary Fig. S9A, purified BRD4 proteins displayed differential binding to E2F1 peptides with distinct post-translational modifications: K117ac/K120ac was bound, while binding to K117/K120ac and K117me1/K120ac was not detectable or much weaker. Additionally, the isolated BD1 domain bound exclusively to the E2F1 K117ac/K120ac peptide, with no detectable binding to the E2F1 K117me1/K120ac peptide (Fig. 5D and Supplementary Fig. S9B). This indicates that the acetylation of K117 and K120 is essential for BRD4 binding. Methylation of K117 counteracts the interaction of E2F1 with the BRD4 BD1 domain by preventing the generation of the K117ac/K120ac diacetylated motif. These data are in line with results showing that PAD4 mediated citrullination of E2F1 in the region 109–127 also disrupts the BRD4 interaction during an inflammatory response [73].

### BRD4 binding is lost with monomethylated E2F1 K117 at cellular level

**Figure 6.**
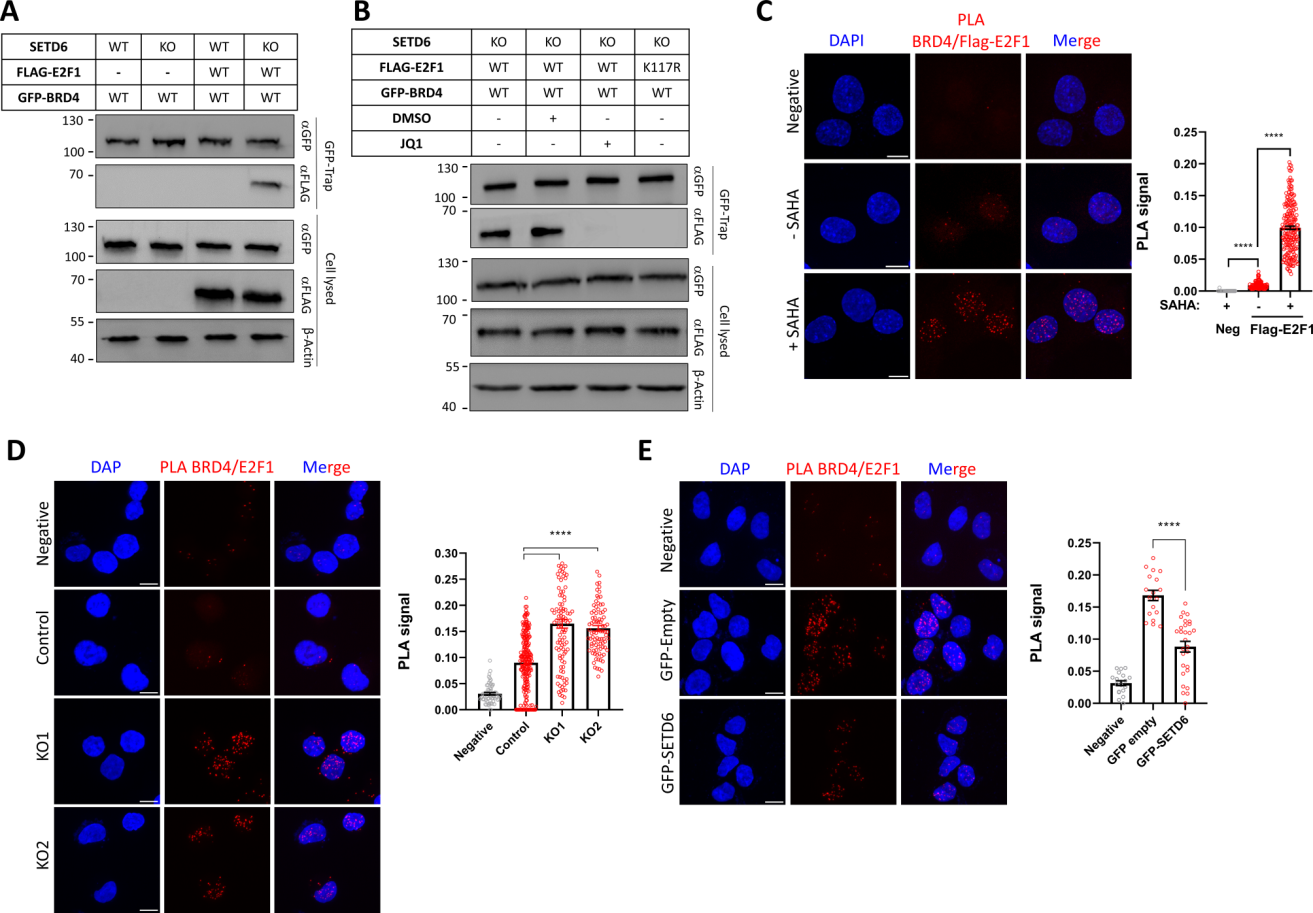
E2F1-BRD4 binding is lost with K117 monomethylated E2F1 in cells. (**A**) GFP-tagged BRD4 (2-477) and Flag-E2F1 (2-437) were transfected into DU145 SETD6 WT and KO cells. The GFP-tagged BRD4 was purified by GFP-trap and analyzed by western-blot with an anti-GFP antibody. Co-purification of Flag-E2F1 was determined by anti-Flag antibody. Equal loading of cell lysate isolated from transfected DU145 SETD6 WT or KO was verified by western-blot analysis against β-actin, GFP, and Flag. (**B**) GFP-tagged BRD4 (2-477) and Flag-E2F1 (2-437) WT or K117R were transfected into in DU145 SETD6 KO cells. Some of the transfected cells were treated with JQ1-Bromodomain-Kac binding inhibitor (5 µM) or DMSO as control. GPF-trap and western-blot analysis was conducted as in panel A. (C–E) Interaction of BRD4 and E2F1 investigated by PLA. All experiments were conducted in DU145 cells. Exemplary microscopy images are shown. Scale bar: 10 µm. PLA signal quantification (PLA dots per nucleus, AU) for each sample is shown on the right. Statistical analysis was performed using Student’s *t*-test in GraphPad (****P < .0001). (**C**) Interaction of endogenous BRD4 and Flag-E2F1 in the absence and the presence of the SAHA deacetylase inhibitor (20 µM) for 5 h (Flag-E2F1). Negative control (Neg) refers to reaction conducted without addition of Flag primary antibody. The interaction of BRD4 and E2F1 was detected and it was shown to be stimulated by increasing acetylation levels after SAHA treatment. Number of analyzed cells: 183, 132, 223. (**D**) Detection of the interaction of endogenous BRD4 and endogenous E2F1 in the presence of 40 µM SAHA for 5 h in DU145 cells (Control) and SETD6 KO cells (KO1 and KO2). Negative control (Negative) refers to reaction conducted without addition of E2F1 primary antibody. Number of analyzed cells: 67, 236, 104, 91. (**E**) Interaction of endogenous BRD4 and endogenous E2F1 in the presence of 40 µM SAHA for 5 h in DU145 cells and with overexpression of GFP (GFP empty) or GFP-SETD6 (GFP-SETD6). Negative control (Negative) refers to reaction conducted without addition of E2F1 primary antibody. Number of analyzed GFP positive cells: 19, 18, 28.

To demonstrate the SETD6-dependent binding of BRD4 and E2F1 in cells, we performed a GFP-trap assay in DU145 prostate cancer cells. This involved single transfections with Flag-E2F1 and GFP-BRD4, as well as co-transfections of both constructs in DU145 SETD6 WT and SETD6 KO cells. Transfection efficiency was verified by western-blot analysis against β-actin as loading control, Flag (for E2F1) and GFP (for BRD4), ensuring equal expression of both proteins (Supplementary Fig. S10A). Western-blot with a specific antibody against E2F1 K117me1 confirmed that E2F1 was specifically methylated in SETD6 WT cells (Supplementary Fig. S10B). After GFP-trap purification of GFP-tagged BRD4, E2F1 was co-purified in SETD6 KO cells, where it was not methylated at K117. However, BRD4 binding was lost in SETD6 WT cells presumably due to the monomethylation of E2F1 at K117 (Fig. 6A and Supplementary Fig. S10C).

To investigate the K117ac dependence of this effect, the E2F1 K117R mutant and the JQ1 inhibitor was used. JQ1 specifically binds to the acetyllysine binding pockets in the BDs of BRD4, thereby disrupting its interaction with acetylated lysine residues [74, 75]. As shown in Fig. 6B, binding of JQ1 to BRD4 disrupted the binding of BRD4 and E2F1 in SETD6 KO cells as indicated by the loss of E2F1 co-purification. Similarly, the K117R mutation disrupted the BRD4–E2F1 interaction supporting the high importance of an acetylated lysine at this position. Additional control experiments suggested that the SETD6 mediated methylation of BRD4 at K99 [14] does not affect the K117me1/ac specificity of the E2F1–BRD4 interaction (Supplementary Fig. S9C and D). This indicates that either inhibiting BRD4 binding to acetylated peptides by JQ1 or disrupting the acetylation of E2F1 and BD1 via the K117R mutation or by K117 methylation in SETD6 WT cells prevents the interaction between BRD4 and E2F1.

To further validate this finding, Proximity Ligation Assay (PLA) was performed to investigate the interaction between BRD4 and E2F1 in DU145 cells. PLA is a sensitive and powerful fluorescence-based technique used to detect and visualize single molecule protein–protein interactions in cells. It relies on the use of two primary antibodies for the two target proteins, each of them conjugated to short DNA strands (PLA probes). If the target proteins are in close proximity (typically within 40 nm), the PLA probes can hybridize and become ligated to form a circular DNA template that is then amplified by rolling circle amplification and detected as a localized fluorescent signal [76]. We first analyzed the interaction of endogenous BRD4 and Flag-E2F1 and observed a strong and specific PLA signal that was further enhanced in the presence of the deacetylase inhibitor SAHA (Fig. 6C). Next, we studied the interaction of endogenous BRD4 and endogenous E2F1 in DU145 cells in the presence of SAHA and again observed a strong PLA signal (Fig. 6D, Control bar), which was further elevated by SETD6 KO (Fig. 6D, KO bars). Finally, we studied the interaction of endogenous BRD4 and endogenous E2F1 in DU145 cells in the presence of SAHA with overexpression of GFP-tagged SETD6 using GFP as control (Fig. 6E) and observed that the E2F1–BRD4 interaction was reduced by SETD6 expression. These data validate the interaction of E2F1 and BRD4, show that it depends on protein acetylation and SETD6 methylation can disrupt it.

### K117 methylation regulates the combined binding of E2F1 and BRD4 at genomic target regions

**Figure 7.**
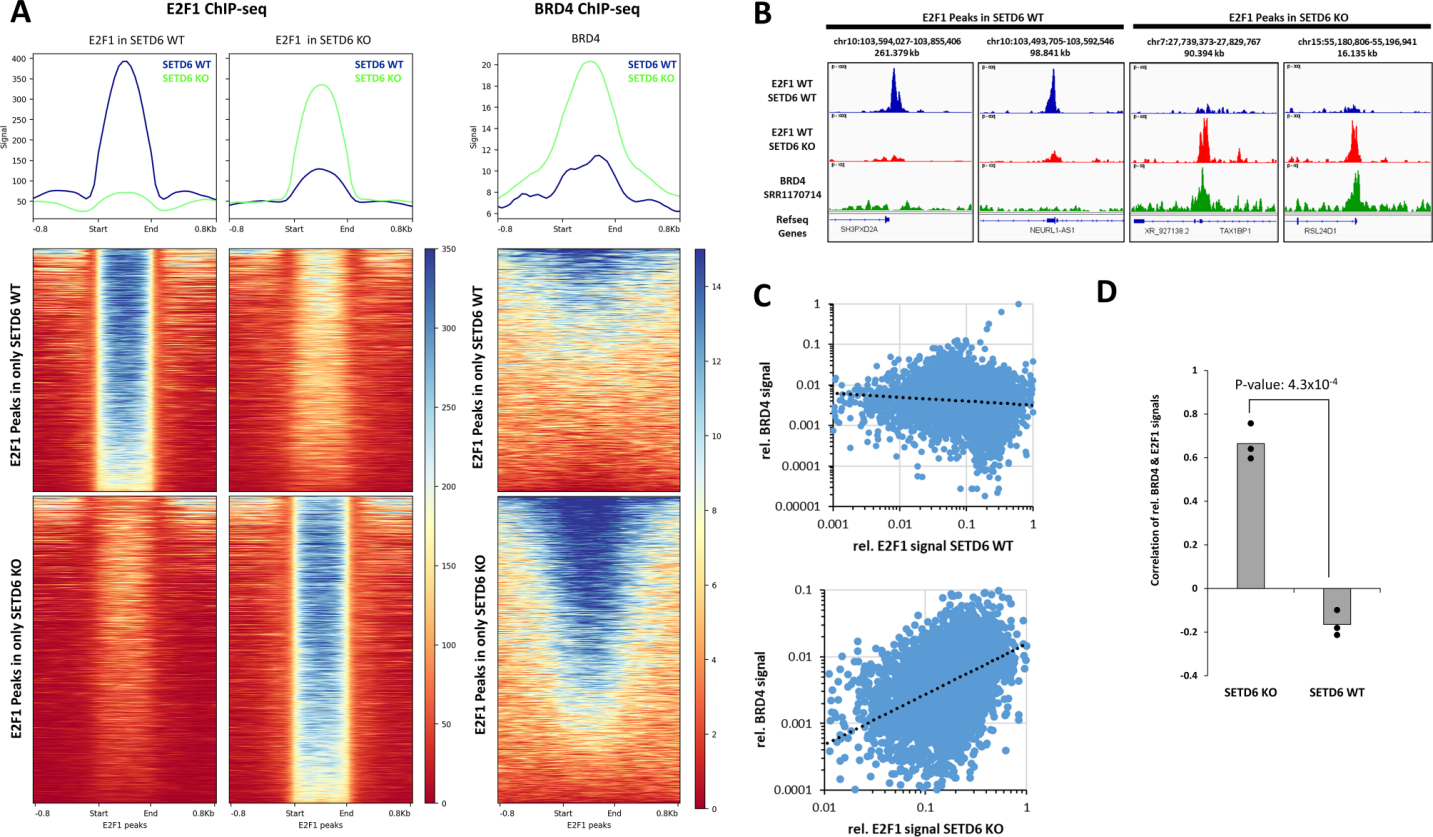
E2F1-BRD4 co-occurence is observed in SETD6 KO but not SETD6 WT cells. (**A**) Heatmap of RPKM-normalized E2F1 ChIP-seq signals at E2F1 peaks (±0.8 kb) showing differential chromatin binding of E2F1 in SETD6 WT and KO cells stably expressing Flag-E2F1. The third heatmap shows BRD4 Chip-seq signals in a prostate cancer cell line (SRR1170714) using the same clustering. See also Supplementary Fig. S11. (**B**) Example browser views showing ChIP-seq of BRD4 (SRR1170714, green) and E2F1 in SETD6 WT and KO cells. See also Supplementary Fig. S12 for additional examples. (**C**) Correlation analysis of E2F1 binding in SETD6 WT and KO cells with the literature BRD4 chromatin binding profile used in panel (A). E2F1 and BRD4 signals were determined in the E2F1 peak regions shown in panel (A) and their correlation was determined. (**D**) Bar-graph showing the slope of the correlation line of BRD4 and E2F1 binding signals in SETD6 WT or KO cells determined using three BRD4 ChIP-seq data sets (datasets SRR1170714, SRR5467129, and SRR5467130). The corresponding analyses are shown in panel (C) and Supplementary Fig. S13. *P*-value determined by two-flanked *t*-test assuming equal variance.

Our data presented so far indicate that BRD4 binds to acetylated E2F1 in SETD6 KO cells, but monomethylation of K117 disrupts this interaction. These data suggest that SETD6 mediated methylation of E2F1 at K117 regulates the co-recruitment of E2F1 and BRD4 to specific genomic regions to regulate selective transcriptional programs. To approach this mechanism from an independent angle, we analysed chromatin binding of E2F1 and BRD4 in cells and examined the effect of SETD6 activity on the co-occurrence of E2F1 and BRD4 binding at chromatin sites. To investigate this, the relation between the E2F1 binding profiles determined here in SETD6 WT and KO cells and BRD4 literature data sets obtained in SETD6 WT prostate cancer cells (SRR1170714 taken from GSE55064 [77], SRR5467129 and SRR5467130 taken from GSE98069 [78]) was analysed. For this correlation analysis, E2F1 binding sites with differential binding in SETD6 WT and SETD6 KO cells were identified and compared with BRD4 binding profiles based on the BRD4 ChIP-seq datasets (Fig. 7A and Supplementary Fig. S11). Strikingly, BRD4 signals were much stronger in genomic regions overlapping with E2F1-bound regions in SETD6 KO cells, containing unmethylated E2F1, than in SETD6 WT cells. To validate these conclusions, ChIP-seq peaks of BRD4 (SRR1170714, green) and E2F1 in both SETD6 WT (blue) and SETD6 KO (red) were visualized using IGV 2.13.1. These ChIP-seq data clearly demonstrate that BRD4 and E2F1 co-occur at genomic loci in SETD6 KO, but not in SETD6 WT cells, supporting our biochemical finding that SETD6 methylation disrupts the binding between BRD4 and E2F1 (Fig. 7B and Supplementary Fig. S12). To further validate this finding, scatter plots of E2F1 and BRD4 binding signals were generated at the E2F1 peak regions from Fig. 7A to examine the correlation of between BRD4 and E2F1 signals in the presence and absence of SETD6 (Fig. 7C and Supplementary Fig. S13). Strikingly, a positive correlation of BRD4 signals was observed with E2F1 signals from SETD6 KO cells, but BRD4 and E2F1 signals had a weak and negative correlation in SETD6 WT. Using different literature BRD4 ChIP-seq data set indicates that this difference is highly significant (Fig. 7D).

### Differential BRD4 binding at genes differentially regulated by E2F1

**Figure 8.**
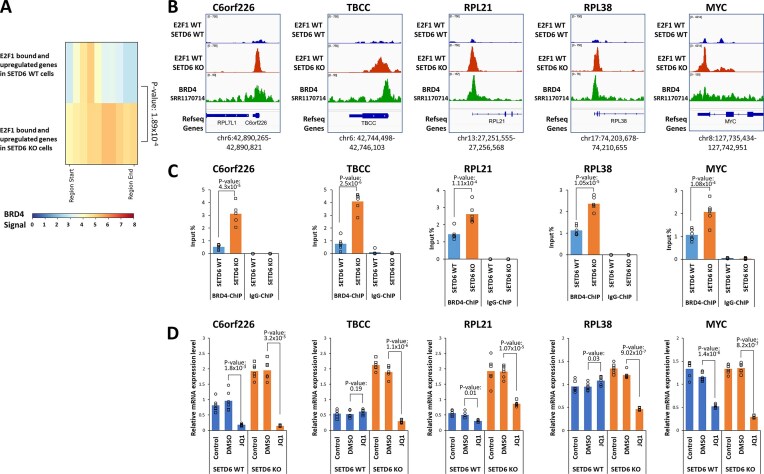
Promoter and enhancer binding of BRD4 at genes preferentially bound by E2F1 and upregulated in SETD6 WT or KO context. (**A**) Average of aggregated BRD4 signals at promoter and enhancer elements of genes preferentially bound by E2F1 and upregulated in SETD6 WT or KO context. Note stronger binding in SETD6 KO cells. *P*-value determined by two-flanked *t*-test assuming equal variance. (**B**) Representative genome browser views showing co-occupancy of BRD4 and E2F1 at five genomic regions in SETD6 KO cells: C6orf226, TBCC, RPL21, RPL38, and MYC. ChIP-seq tracks were visualized using IGV (version 2.13.1) displaying BRD4 (SRR1170714, green), E2F1 in SETD6 WT (blue), and E2F1 in SETD6 KO (red) DU145 cells. (**C**) ChIP from SETD6 WT and KO DU145 cells performed using a BRD4-specific antibody to enrich BRD4-bound chromatin fragments. IgG was used as a negative control to assess the specificity of the immunoprecipitation. BRD4 occupancy was evaluated by qPCR at the same loci as shown in panel (B). Two independent biological replicates with three technical repeats were performed. Statistical significance was determined using a two-tailed *t*-test assuming equal variance. The negative controls RPL21 and RPL28 did not yield a detectable signal. Note the elevated BRD4 binding in SETD6 KO context. (**D**) RT-qPCR analysis of the relative expression of the five target genes (Supplementary Table S2) shown in panel (B) in untreated SETD6 WT and KO DU145 cells (control) as well as after addition of DMSO and JQ1. Note the strong effect of JQ1 on gene expression in SETD6 KO cells.

Finally, we wanted to find out, whether the disruption of the BRD4-E2F1 interaction by SETD6 also contributes to the differential binding and regulation of genes by E2F1 in SETD6 WT or KO context shown in Figs. 2 and 3. To this end, the promoter and enhancer regions of all affected genes were retrieved using the EnhancerAtlas 2.0 database (http://www.enhanceratlas.org/) [31] and the corresponding BRD4 signals were derived from the BRD4 ChIP-seq data set SRR1170714 (taken from GSE55064 [77]). Next, the profile of the average BRD4 signals of the promoters and enhancers of the 40 genes with strong E2F1 binding and upregulation in SETD6 WT was plotted showing a clear enrichment of BRD4 signal in the center of the elements as expected as BRD4 is well-known to bind these genomic elements (Fig. 8A). The same was observed with the 210 genes with strong E2F1 binding and upregulation in SETD6 KO, but in this case the BRD4 signals were much stronger, which is in agreement with our finding that the E2F1-BRD4 interaction is stronger in SETD6 KO cells.

Next, we conducted ChIP-qPCR and RT-qPCR experiments to analyze BRD4 chromatin binding and its effect on gene expression in SETD6 WT and KO DU145 cells. For this, candidate genes were selected from our list of genes showing stronger E2F1 binding and upregulation in SETD6 KO conditions, and from a previous publication reporting combined effects of E2F1 and BRD4 on transcription [14]. The required panel of qPCR amplicons (Supplementary Table S1) could be successfully established for C6orf226 and TBCC from our list as well as RPL21, MYC, and RPL38 from [14]. All loci showed binding of E2F1 mainly in SETD6 KO cells and colocalization with published BRD4 ChIP-seq data (SRR1170714 [79]) (Fig. 8B). Next, BRD4 binding was investigated in our SETD6 WT and KO DU145 cell by ChIP-qPCR. Our data document that BRD4 occupancy at all tested loci was significantly higher in SETD6 KO cells compared to SETD6 WT cells, indicating that SETD6 negatively regulates BRD4 recruitment to E2F1 target genes (Fig. 8C). No signal was detected in the IgG controls (Fig. 8C) and no enrichment was detected at a negative control region (Supplementary Fig. S14).

Finally, we investigated whether expression of these genes in our SETD6 WT and KO DU145 cells depends on the binding of BRD4 to acetylated partner proteins. For this, RT-qPCR experiments were conducted in the absence and presence of JQ1. Our data demonstrate that expression of all target genes was strongly reduced in SETD6 KO cells, suggesting that under these conditions, JQ1 can disrupt an existing BRD4–E2F1 interaction. In SETD6 WT cells, JQ1 showed no or weak effects at three genes (TBCC, RPL21, RPL38). In case of C6orf226, gene expression and JQ1 effects were weaker in SETD6 WT cells than in KO cells. Only at the MYC locus, JQ1 effects were comparable in SETD6 WT and KO cells, suggesting that BRD4 is important for its expression also in SETD6 WT cells where it presumably interacts with another acetylated protein than E2F1.

In summary, these results confirm our findings that SETD6 methylation of E2F1 at K117 disrupts the interaction of BRD4 and E2F1 leading to a loss of the correlated chromatin binding of both factors. Moreover, these data clearly document that regulation of the E2F1–BRD4 interaction contributes to the differential chromatin binding and gene regulation of E2F1 detected in our SETD6 WT and KO DU145 cells. Based on this analysis, we conclude that the regulation of the E2F1–BRD4 interaction is an important function of SETD6 leading to differential chromatin binding and gene regulation of E2F1 under SETD6 WT and KO conditions.

## Discussion

Lysine methylation is a widespread protein PTM that regulates critical protein properties including protein activity, stability, and protein/protein interactions [70]. Lysine methylation of chromatin proteins including transcription factors has been implicated in various steps of the initiation and progression of cancer [80, 81]. The SETD6 PKMT is known as lysine mono-methyltransferase that methylates a wide spectrum of substrate proteins with important roles in normal cell physiology and in cancer development [10]. In our previous study, we identified E2F1 K117 as a novel SETD6 substrate in prostate cancer cells [25]. E2F1 is a transcription factor with important roles in prostate cancer [82]. Here, we explored how SETD6 methylation influences the E2F1 activity in prostate cancer cells.

In combined ChIP-seq and RNA-seq experiments, we observed differential transcriptional regulation in WT SETD6 WT or SETD6 KO prostate cancer cells expressing Flag-E2F1 (Figs 2 and 3). ChIP-seq showed a differential E2F1 binding in SETD6 WT versus SETD6 KO cells, with distinct clusters that indicate methylation-dependent chromatin binding of E2F1. Additionally, RNA-seq revealed significant changes in gene expression with 1081 genes upregulated by E2F1 WT in SETD6 KO cells and 257 genes upregulated by E2F1 WT in SETD6 WT. Integration of both analyses led to the discovery of 40 genes that are effectively bound and upregulated by E2F1 in SETD6 WT cells. The differential effect of E2F1 WT and K117R considered in our workflow suggests that these genes are upregulated by K117 methylated E2F1. These genes participate in the regulation of prostate gland development, morphogenesis, apoptosis, and negative regulation of cell adhesion and migration. On the other hand, 210 genes showed strong E2F1 binding and upregulation in SETD6 KO suggesting that they are upregulated by unmethylated E2F1. These genes were connected to negative regulation of cell death and mitochondrial regulation. These distinct roles of E2F1 in the presence and the absence of SETD6 might explain previous findings pointing toward a conflicting role of E2F1 in prostate cancer [82], because in the light of our findings the effects of E2F1 on tumor progression depend on the SETD6 expression level of the cancer cell. In future, it will be interesting to conduct more functional assays and further investigate the role of SETD6 and E2F1 K117 methylation in the control of cell migration, prostate gland differentiation and apoptosis.

These data indicate that K117 methylation of E2F1 controls its transcriptional output. In this context, it is of relevance that we previously showed that E2F1 also binds to the SETD6 promoter and stimulates SETD6 expression, and this effect was enhanced by K117 methylation [25]. This creates a positive feedback in the SETD6/E2F1 system restricting it to two stable states:

- State 1: No/low expression of SETD6, no methylation of E2F1, no/low stimulation of SETD6 expression by E2F1
- State 2: High expression of SETD6, methylation of E2F1, strong stimulation of SETD6 expression by E2F1 K117me1

**Figure 9.**
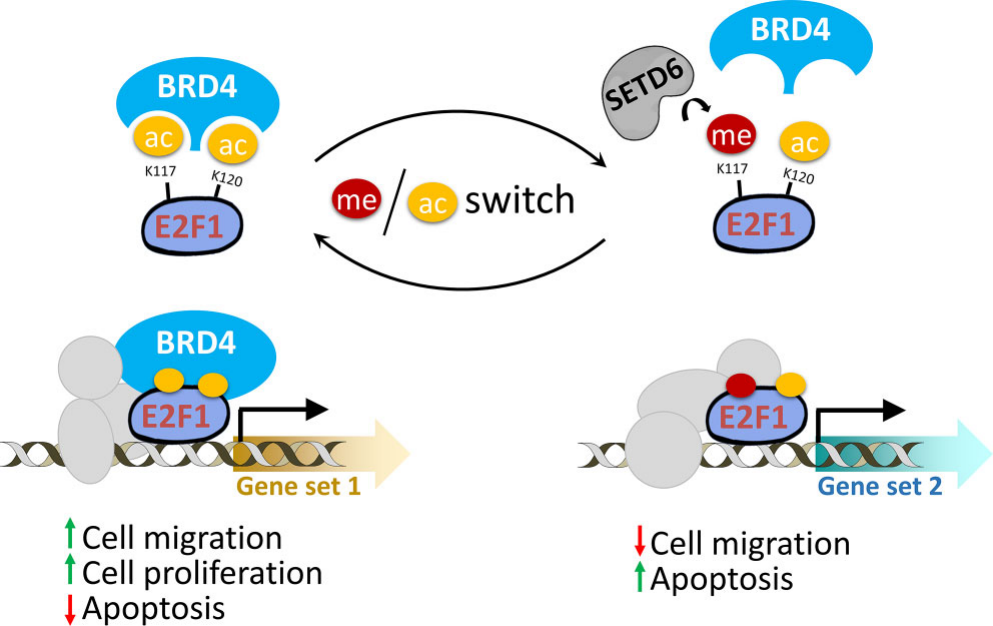
Summary of the results of this study. SETD6 monomethylates E2F1 at K117. This methylation disrupts the E2F1–BRD4 interaction leading to different target loci being bound by both factors. In the absence of K117 methylation, E2F1 is acetylated at K117 and K120 leading to BRD4 binding and a concerted engagement of both protein at genomic target sites. As a consequence, methylated and unmethylated E2F1 regulates distinct gene sets in prostate cancer cells.

We now show in this work that switching the K117 methylation state in E2F1, changes its transcriptional outcome from the expression of gene related to prostate gland development, morphogenesis, and negative regulation of cell adhesion and migration (E2F1 K117me1) to genes related to negative regulation of cell death and mitochondrial regulation (E2F1 with unmethylated K117). When combined our data suggest that E2F1 K117 methylation mediates the conversion of one cellular state into another one in a switch-like process triggered by SETD6 (Fig. 9).

A previous study has reported that increasing E2F1 expression levels are associated with tumor growth and cell survival rate of prostate cancer [33]. Additionally, mitochondria have a potential role in energy production, different metabolic processes, and apoptosis. Dysregulation of mitochondrial function has been observed in several types of cancer, including prostate cancer. Problems in mitochondrial functionality can affect energy metabolism, ROS production, and apoptotic pathways with potential effects on cell proliferation, growth, cell death processes, and metastasis [83, 84]. For this reason, we performed apoptosis and cell proliferation assays showing that SETD6 KO in prostate cancer cells reduced apoptosis and induced cell proliferation that could be partially connected to K117 methylation of E2F1.

Following the intriguing findings from our ChIP-seq and RNA-seq analyses, we also aimed to discover the detailed molecular mechanism of the SETD6 dependent distinct cellular regulation by methylated and unmethylated E2F1. In this context, it is of relevance that E2F1 is known to be acetylated at K117 and K120. E2F1 acetylation stabilizes the protein in response to DNA damage and may target it to pro-apoptotic genes [75]. Acetylation of E2F1 at lysine residues K117 and K120 creates a strong binding motif for BD1 of BRD4 [24] that is disrupted by E2F1 K117 methylation. We show here with overexpressed and endogenous proteins that this interaction depends on E2F1 K117 acetylation and monomethylation of K117 by SETD6 disrupts E2F1 binding to BRD4 in vitro and at cellular level. These findings reveal a molecular mechanism of how K117 methylation by SETD6 can directly affect E2F1 function and gene regulation (Fig. 9). In addition, acetylation of E2F1 has been shown to create binding sites for the BDs of the p300/KAT3B and CBP/KAT3A acetyltransferases and that this interaction controls the recruitment of p300 and CBP to chromatin sites [23]. Future work is needed to decipher the potential effects of SETD6 methylation of K117 on the interaction of E2F1 with these proteins and its cellular effects. Regulation of the binding of other BD containing proteins to E2F1 by K117 methylation could explain our finding that not all of the differentially bound and regulated genes identified in our study showed differential BRD4 signals. In addition, the crosstalk of K117 methylation with other arginine methylation and lysine acetylation events of E2F1 nearby must be addressed in future work.

## Conclusions

SETD6 monomethylates E2F1 specifically at lysine residue K117. We studied chromatin binding and gene regulation by methylated and unmethylated E2F1 and identified non-overlapping sets of selectively affected genes. Forty genes were strongly bound by E2F1 and upregulated in SETD6 WT cells in the context of E2F1 WT, while a distinct set of 210 genes were strongly bound by E2F1 and upregulated in SETD6 KO cells. Many genes in both sets are connected to cancer related phenotypes. In agreement with this, SETD6 KO cells revealed reduced apoptosis and enhanced cell proliferation. Methylation of K117 mechanistically prevents acetylation at this site, thereby inhibiting the interaction of E2F1 with BD1 of BRD4. As a result, chromatin binding of K117 methylated E2F1 does not show correlation with BRD4 binding. Hence, SETD6 methylation at E2F1 K117 directly regulates the interaction of E2F1 and BRD4, which can explain the differential effects of methylated and unmethylated E2F1. Increased activity of methylated E2F1 at the SETD6 promoter generates a positive feedback loop allowing cells to switch between high and low SETD6 activity states. In a more general context, our data suggest that K117 methylation/acetylation of E2F1 may represent an important methyl/acetyl switch leading to a wide range of potential cellular effects triggered by monomethylation of a single lysine residue. To further explore the signalling capacities of E2F1 K117 methylation, it will be interesting to investigate in future research, whether there is active enzymatic removal of the K117 methylation by one of the known KDMs or whether this depends on E2F1 protein turnover.

## Supplementary Material

gkaf1513_Supplemental_Files

## Data Availability

The source data to Fig. 1B and D are provided in Supplementary Data S1. The E2F1 ChIP-seq FastQ files and analyzed BigWig files are provided at GEO GSE286020 dataset. The RNA-seq FastQ files and analyzed read tables are provided at GEO GSE286198 dataset.
