# Supplementary material for: E2F1 K117 methylation by SETD6 disrupts BRD4–E2F1 binding and modulates E2F1 chromatin binding and gene regulation in prostate cancer cells
Source: Nucleic Acids Res. 2026 Jan 15;54(2):gkaf1513. doi: 10.1093/nar/gkaf1513 (PMC12808549; doi:10.1093/nar/gkaf1513)
Supplement: gkaf1513_Supplemental_Files [file gkaf1513_supplemental_files.zip › SETD6_E2F1_BRD4_rev2_suppl_2.pdf]

E2F1 K117 methylation by SETD6 disrupts BRD4-E2F1 binding and modulates E2F1 chromatin binding and gene regulation in prostate cancer cells

Supplementary information

Supplementary figures

Supplementary Figure 1: Characterization of the cell lines used in this study

Supplementary Figure 2: ChIP-qPCR experiments showing the chromatin binding of Flag-E2F1 in SETD6 WT or KO DU145 prostate cancer cells

Supplementary Figure 3: Correlation between different ChIP-seq and input data sets obtained from Flag-E2F1 expressing SETD6 WT or KO DU145 prostate cancer cells

Supplementary Figure 4: Examples of binding profiles of E2F1 in SETD6 WT or KO DU145 prostate cancer cells

Supplementary Figure 5: Correlation of the binding sites of E2F1 in SETD6 WT cells determined here and in published data sets

Supplementary Figure 6: Heatmaps of E2F1 binding sites in SETD6 WT or KO DU145 prostate cancer cells

Supplementary Figure 7: Clustering and correlation of RNA-seq data obtained in SETD6 WT and KO DU145 prostate cancer cell line expressing Flag E2F1 WT or E2F1 K117R

Supplementary Figure 8: Additional data related to Figure 4

Supplementary Figure 9: Additional peptide SPOT binding assays related to Figure 5

Supplementary Figure 10: Quality control of the Western-blots used to analyze BRD4-E2F1 binding in SETD6 WT cells

Supplementary Figure 11: Additional heatmaps of E2F1 and BRD4 Chip-seq data related to Figure 7A

Supplementary Figure 12: Exemplary browser views showing co-occurrence of BRD4 and E2F1 peaks in SETD6 KO cells, but only E2F1 peaks in SETD6 WT cells

Supplementary Figure 13: Correlation analysis of BRD4 and E2F1 binding in SETD6 WT and KO cells
Supplementary Figure 14: Quality control of the BRD4 ChIP shown in Figure 8

Supplementary tables

Supplementary Table 1: Primers used in ChIP-qPCR assays

Supplementary Table 2: Primers used in RT-qPCR assays

Supplementary Table 3: List of genes strongly bound and upregulated by E2F1 WT but not E2F1 E117K in WT SETD6 DU145 cells

Supplementary Table 4: List of genes strongly bound and upregulated by E2F1 WT but not E2F1 E117K in KO SETD6 DU145 cells

Other Supplementary materials

Supplementary Data Table 1 (provided as separate file): Source data of Figure 1B.

Supplementary references

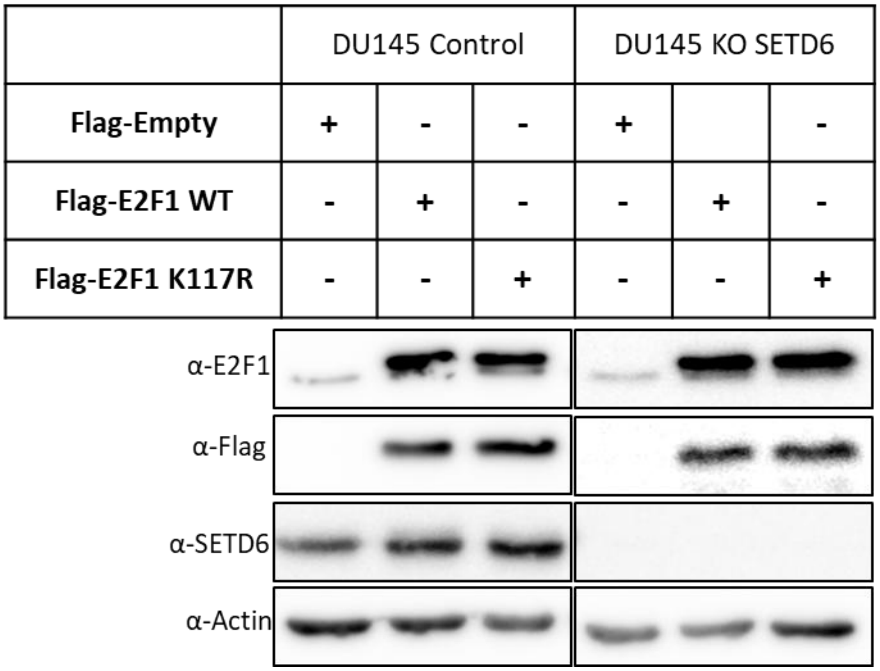

Supplementary Figure 1: Characterization of the cell lines used in this study. The generation of SETD6 KO DU145 cell lines with or without Flag-E2F1 WT or K117R mutant overexpression has been described in Kublanovsky et al. (2023) (1). Protein lysates from these cells were analyzed by Western blotting using anti-Flag and anti-E2F1 antibodies to detect exogenous and total E2F1 expression. SETD6 protein levels were determined using a SETD6-specific antibody to confirm knockout efficiency. Actin was used as a loading control for normalization. These results confirm successful overexpression of Flag-E2F1 WT and mutant at comparable amounts and validate the loss of SETD6 in the KO cells.

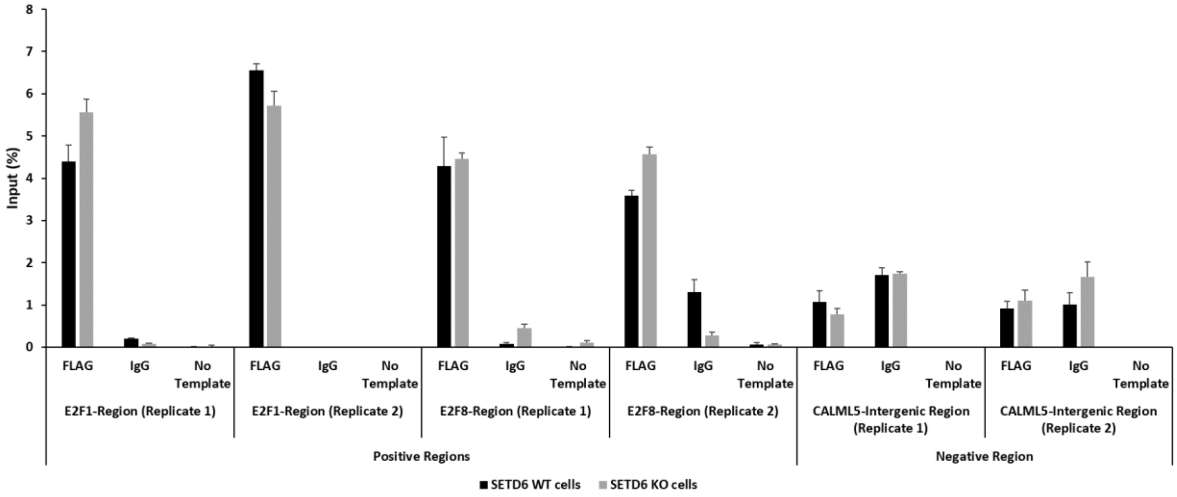

Supplementary Figure 2: ChIP-qPCR experiments showing the chromatin binding of Flag-E2F1 in SETD6 WT or KO DU145 prostate cancer cells. The ChIP experiment was performed by using a Flag antibody to precipitate Flag-E2F1 from sonicated chromatin. For normalization, all fold enrichment values were determined by comparison with input chromatin controls using input percentages of 10%, 2%, and 0.4%. As negative control, an IgG antibody was used as to ensure specificity and enrichment of the immunoprecipitation. As negative control of the PCR, no template reactions were included. The E2F1 and E2F8 loci served as positive controls for E2F1 binding, while the CALML5 region was used as a negative E2F1 binding control region.

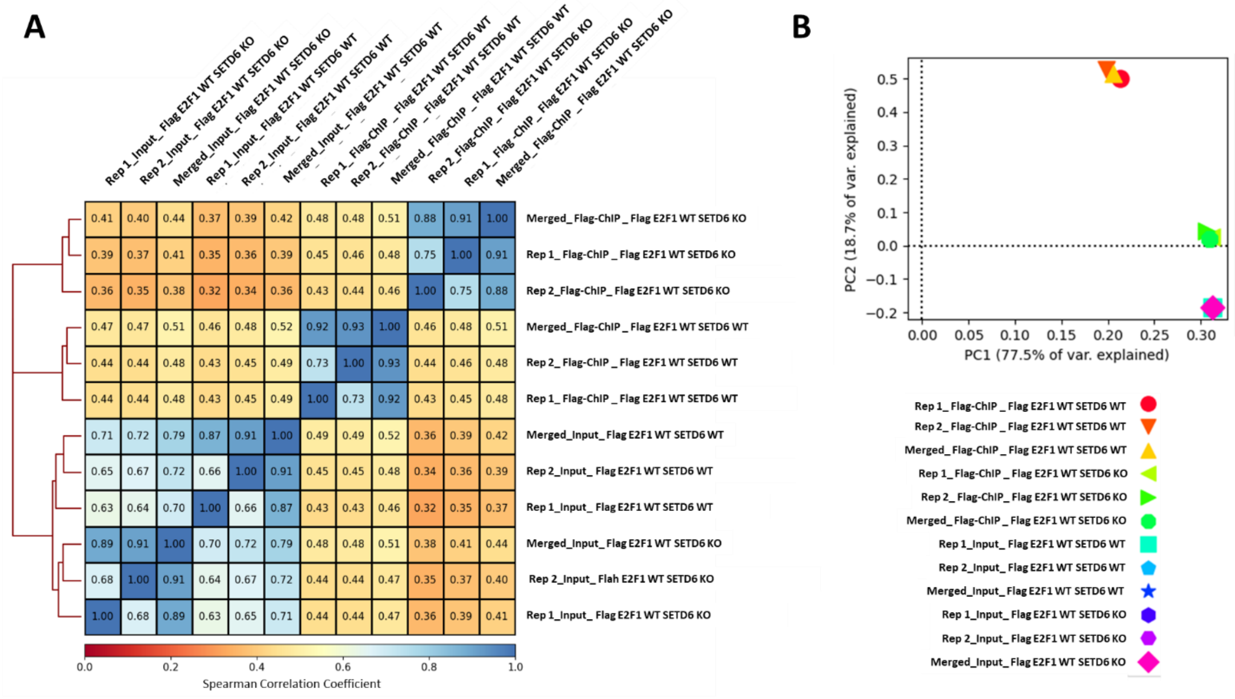

Supplementary Figure 3: Correlation between different ChIP-seq and input data sets obtained from Flag-E2F1 expressing SETD6 WT or KO DU145 prostate cancer cells. A) All data were acquired in biological duplicates, replicate 1 (Rep 1) and replicate 2 (Rep 2) and merged data sets were prepared as well (Merged). The heatmap shows correlation coefficients for signals calculated in 5000 bp bins by plotCorrelation using Spearman correlation. **B)** Principal component analysis (PCA) plot generated from ChIP-seq data of Flag E2F1 expressed in SETD6 WT and SETD6 KO DU145 prostate cancer cell line using MultiBigwigSummary and PlotPCA (all modules were form Galaxy Version 3.5.4+galaxy0).

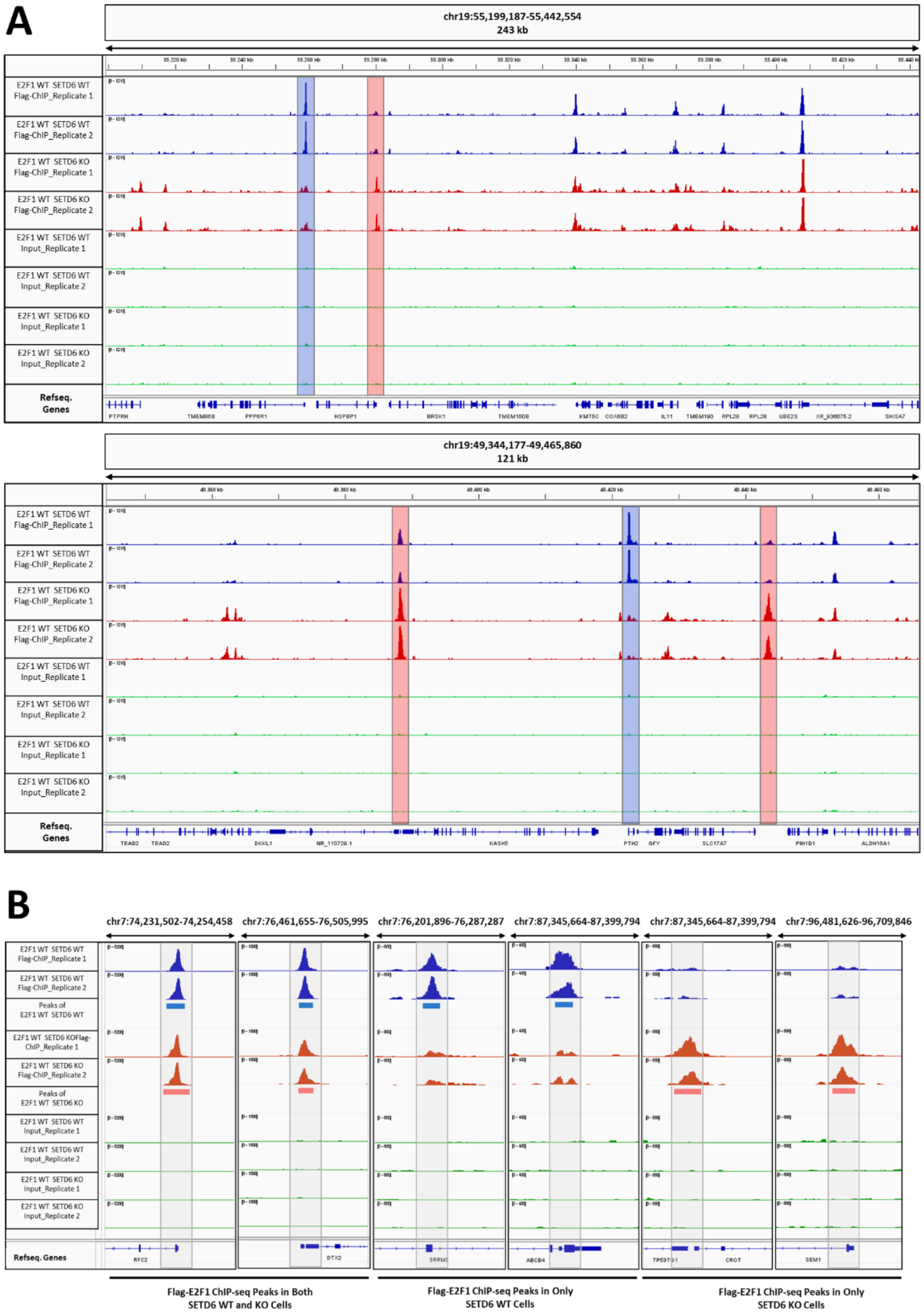

Supplementary Figure 4: Examples of binding profiles of E2F1 in SETD6 WT or KO DU145 prostate cancer cells. Integrative Genomics Viewer (IGV) was used to visualize E2F1 tracks in SETD6 WT (blue) and SETD6 KO cells (red), and input samples (green) after alignment to the human hg38 genome. The genomic regions are indicated. **A)** Differential binding regions are highlighted with red and blue boxes. **B)** Annotated peaks are indicated to demonstrate reliable peak calling.

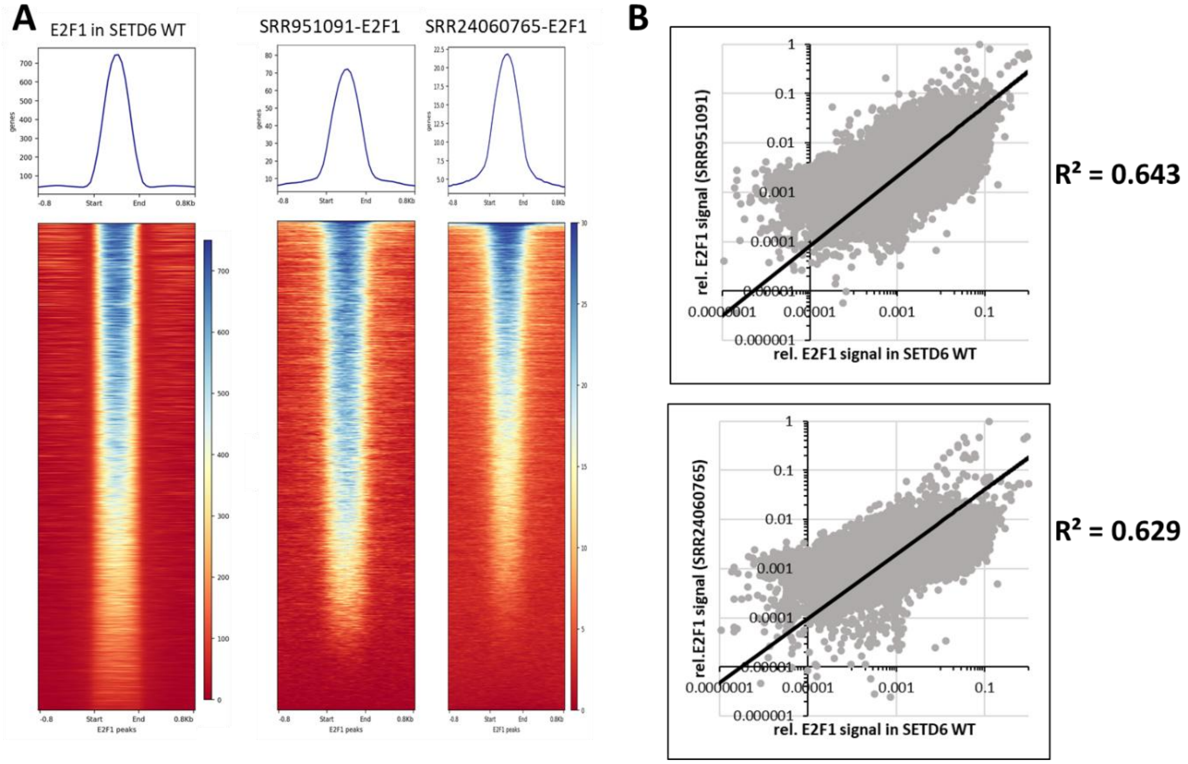

Supplementary Figure 5: Correlation of the binding sites of E2F1 in SETD6 WT cells determined here and in published data sets. A) Heat map of E2F1 RPKM-normalized ChIP-seq signals at E2F1 peaks (± 0.8 kb) in SETD6 WT cells and in literature datasets obtained in prostate cancer cells (SRR951091, SRR24060765). **B)** Correlation of E2F1 binding signals obtained here with corresponding signals in literature data sets.

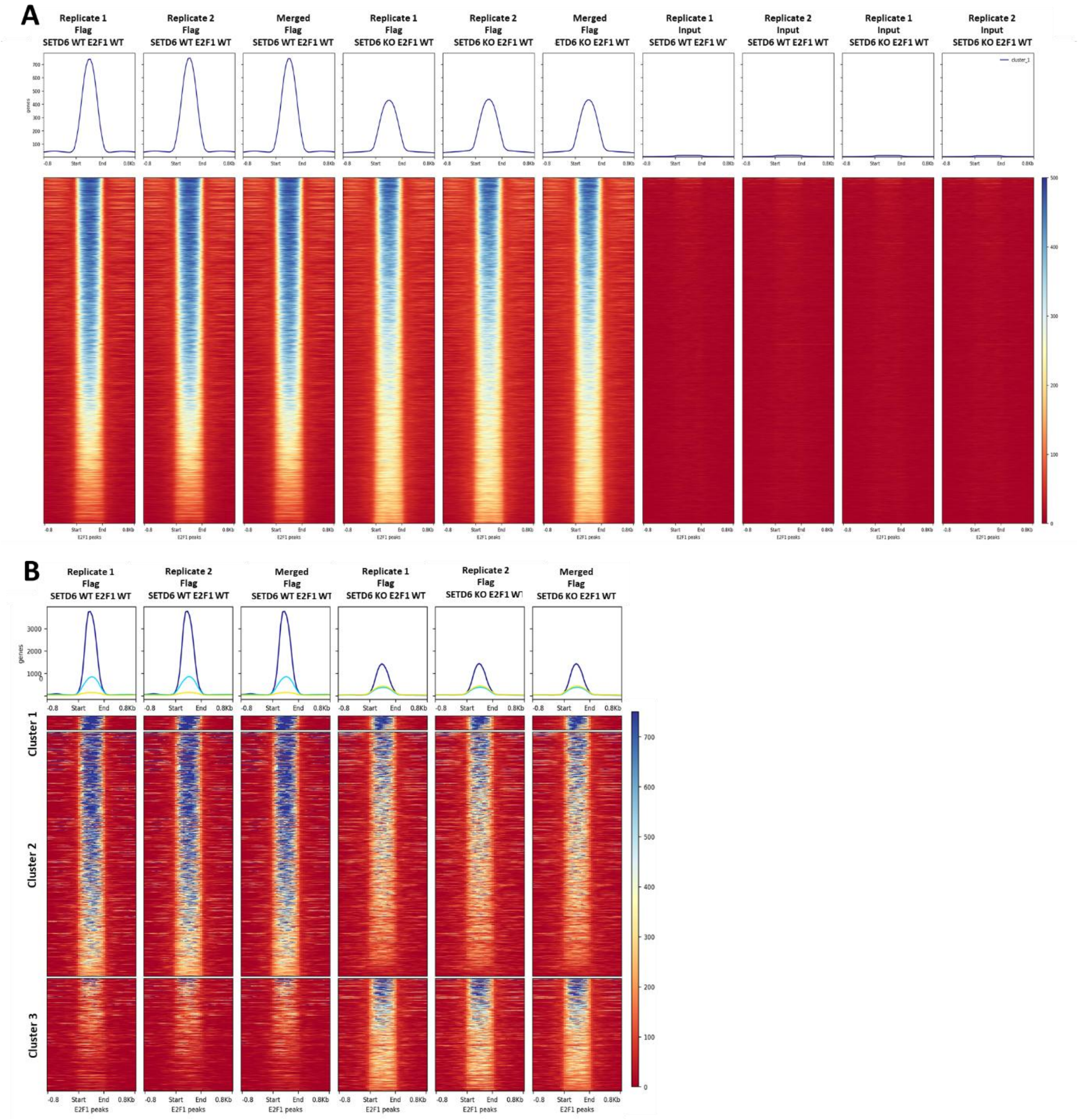

Supplementary Figure 6: Heatmaps of E2F1 binding sites in SETD6 WT or KO DU145 prostate cancer cells. A) E2F1 peaks were annotated in all data sets using ComputeMatrix and PlotHeatmap (both Galaxy Version 3.5.4+galaxy0). Then, all peaks were merged and heatmaps of RPKM-normalized ChIP-seq signals were prepared showing regions of 0.8 kb up- and downstream of the E2F1 binding sites. **B)** The heatmap of E2F1-binding regions shown in panel A was clustered by the k -means clustering into three groups, one with similar binding of E2F1 in SETD6 WT and KO cells (cluster 1, 596 regions), one with preferential binding in SETD6 WT cells (cluster2, 10377 regions) and one with preferential binding in SETD6 KO cells (cluster 3, 4799 regions). Merged images are shown in Figure 1B.

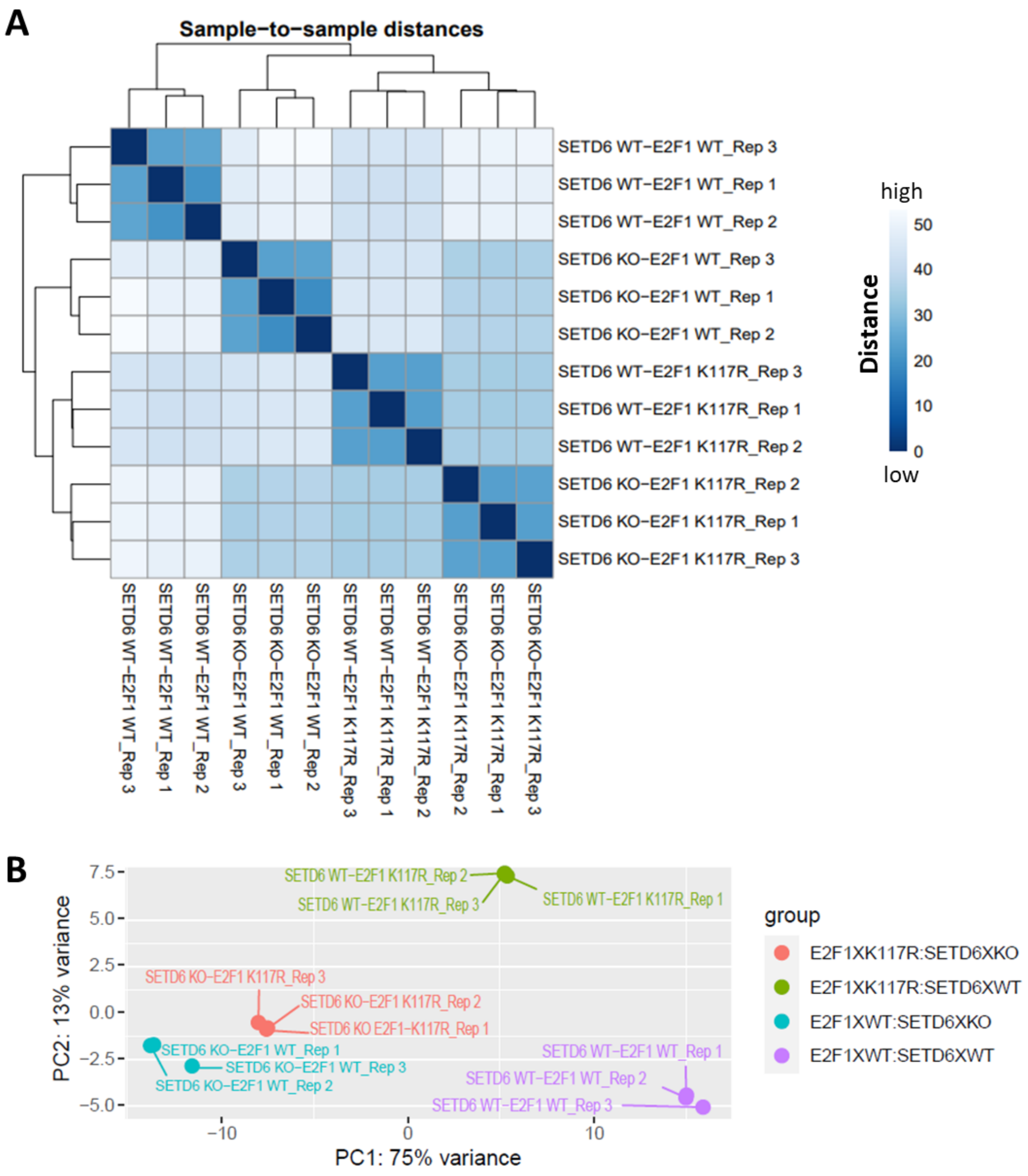

Supplementary Figure 7: Clustering and correlation of RNA-seq data obtained in SETD6 WT and KO DU145 prostate cancer cell line expressing Flag E2F1 WT or E2F1 K117R. A) Heatmap of the sample distance matrix prepared by DESeq2. RNA-seq data is indicated for each sample. Dark-blue represents strong correlations, light-blue represents low correlations. **B)** Principal component analysis (PCA) plot of RNA-Seq samples generated by DESeq2. The two top components, PC1 and PC2, explaining 75% and 13% percentages of variation in the RNA-Seq data, are shown.

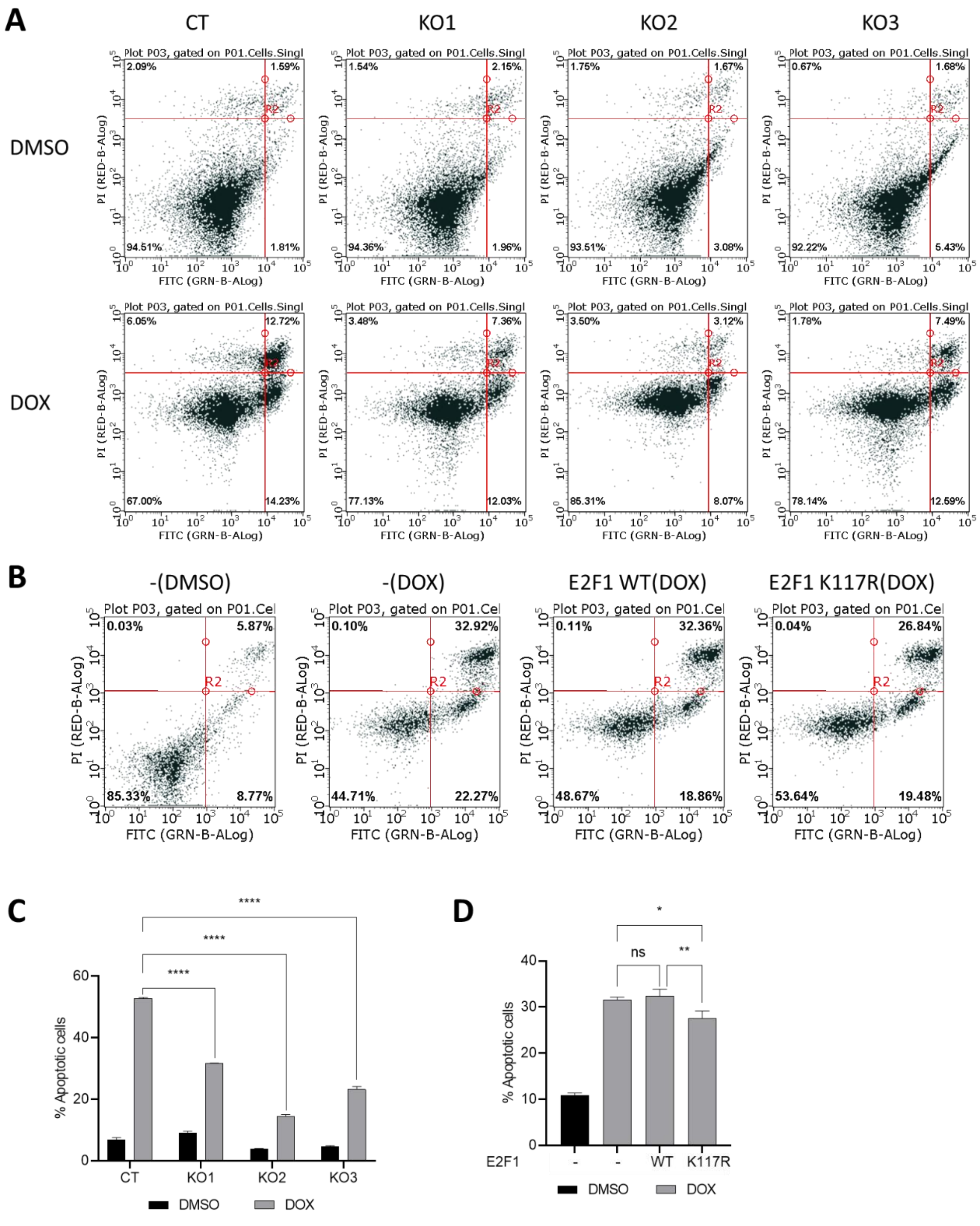

Supplementary Figure 8: Additional data related to Figure 4. A) Exemplary cytometry primary data related to the apoptosis assays shown in Figure 4B. **B)** Exemplary cytometry primary data related to the apoptosis assays shown in Figure 4C. **C)** Results of a biological replicate of the experiment shown in Figure 4B. **D)** Results of a biological replicate of the experiment shown in Figure 4C. The experiments in panel C and D were conducted as described in the legend of Figure 4 except that 5 µM DOX was used.

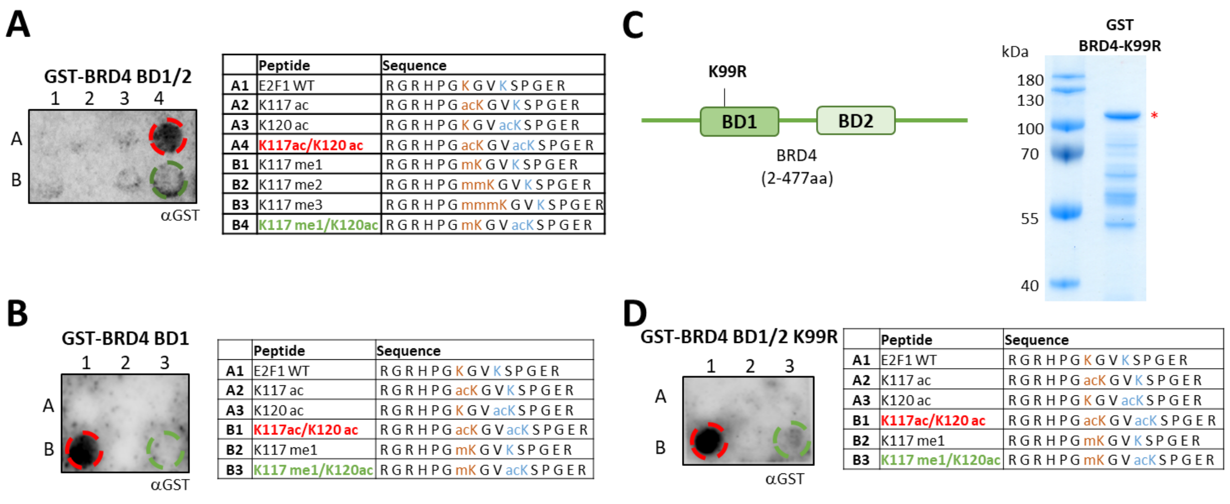

Supplementary Figure 9: Additional peptide SPOT binding assays related to Figure 5. A) Additional peptide array investigating the binding of the GST-BRD4 BD1/2 to the modified E2F1 peptides containing different combinations of unmodified, acetylated, and methylated K117 and K120. Peptide SPOT arrays were incubated with 1 nM GST-BRD4 BD1/2, and binding was subsequently detected using a GST-specific antibody. Strong BRD4 binding is observed to E2F1 K117ac/K120ac (red highlight) while the replacement of the acetyl group at K117 by a methyl groups disrupts the binding (green highlight). **B)** Same as in A but for GST-BRD4 BD1. **C)** Previous work has shown that SETD6 methylates BRD4 at K99 which is located in the BD1 domain (2). Therefore, we investigated whether BRD4 K99R binds K117me1/K120ac E2F1 peptides differently than BRD4 WT. The image shows a Coomassie BB stained 12% SDS gel of the purified GST tagged BRD4 BD1/2 K99R (2-477aa) protein marked with red asterisk. **D)** Binding of GST-BRD4 BD1/2 K99R to modified E2F1 peptides analyzed as shown in panel B. The peptide array was incubated with 50 nM GST-BRD4 BD1/2 K99R and binding was detected using a GST-specific antibody. These data shown that the purified BRD4 K99R mutant proteins exhibited specific binding to K117ac/K120ac, similar to BRD4 WT suggesting that K99 (and in extrapolation its monomethylation) does not play a role in E2F1 K117ac/K120 recognition.

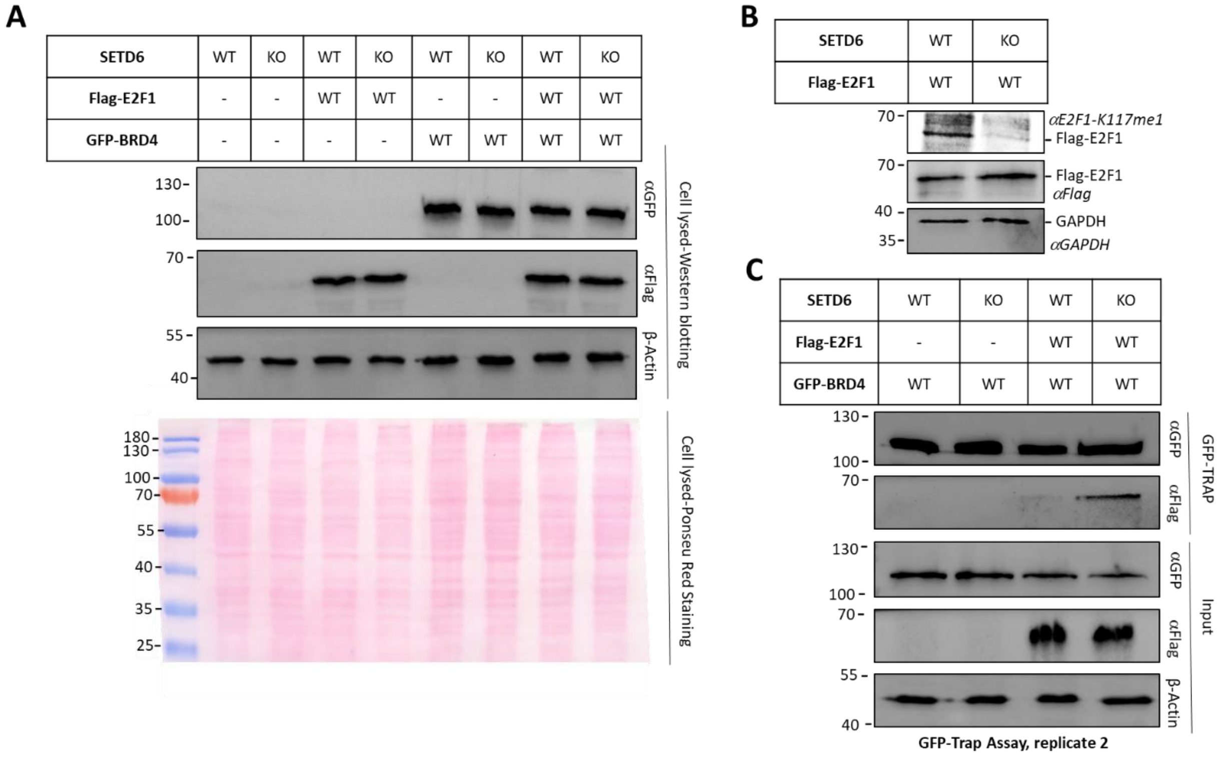

Supplementary Figure 10: Quality control of the Western-blots used to analyze BRD4-E2F1 binding in SETD6 WT cells. A) Single GFP-tagged BRD4 (2–477) and Flag E2F1 (2-437) or both constructs were transfected into in SETD6 WT and KO DU145 cells. Equal loading was verified by Western-blot against β-actin and by Ponceau Red staining. Expression of GFP-tagged BRD4 and Flag-tagged E2F1 was determined by GFP and Flag antibodies. **B)** After transfection of Flag-E2F1 in SETD6 WT cells, monomethylated E2F1 at K117 was determined by specific anti-E2F1me1 antibody. GAPDH and Flag were used as loading control. **C)** Replicate of the GFP-trap assay shown in Figure 6A to detect binding of the E2F1-BRD4 at cellular level.

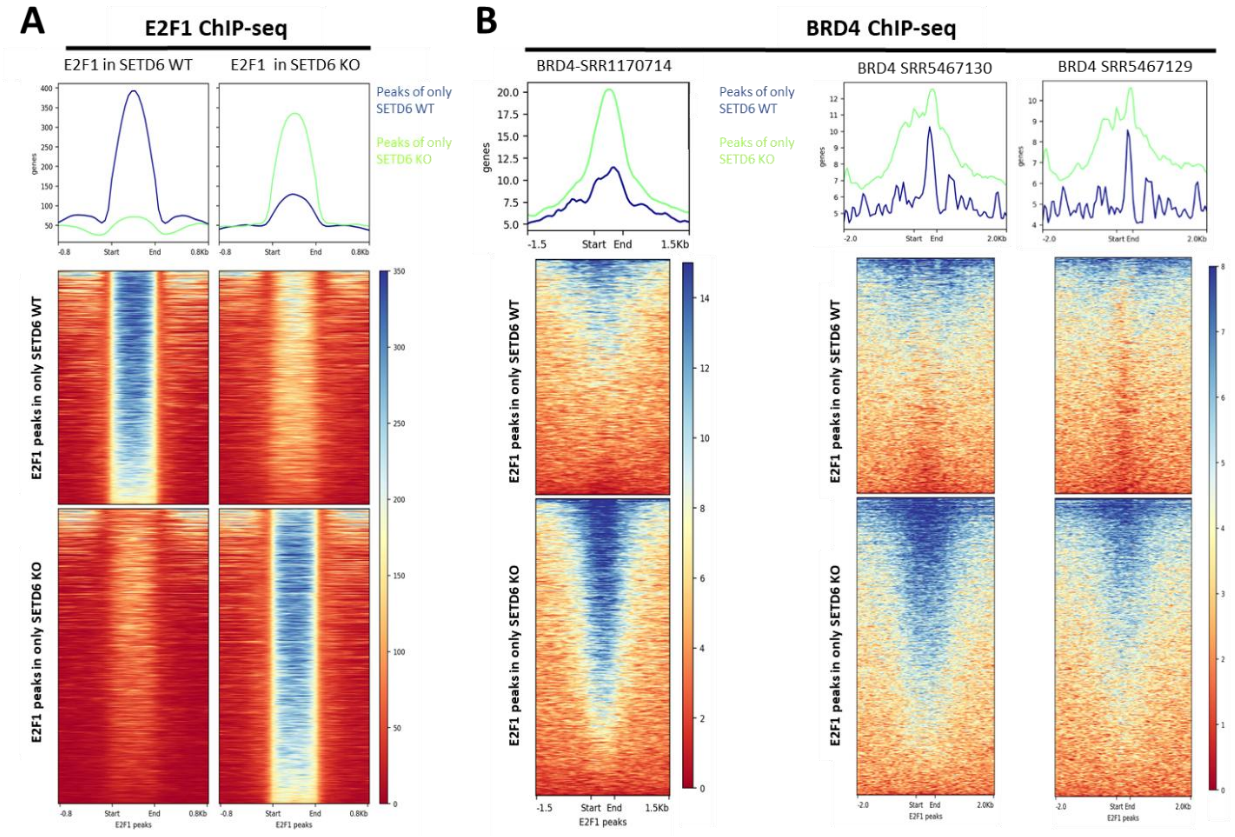

Supplementary Figure 11: Additional heatmaps of E2F1 and BRD4 Chip-seq data related to Figure 7A. A) Heat map of RPKM-normalized E2F1 ChIP-seq signal at E2F1 peaks (± 0.8 kb) showing differential binding of E2F1 in SETD6 WT and KO cells stably expressing Flag-E2F1 (taken from Figure 7A). **B)** BRD4 Chip-Seq signals from a prostate cancer cell lines are shown using the same clustering. The SRR1170714 sample is already shown in Figure 7A, and reproduced here for comparison. The SRR1170730 and SRR5467129 samples are provided here as additional examples. In all cases, stronger BRD4 signals at E2F1 peaks were observed in SETD6 KO cells.

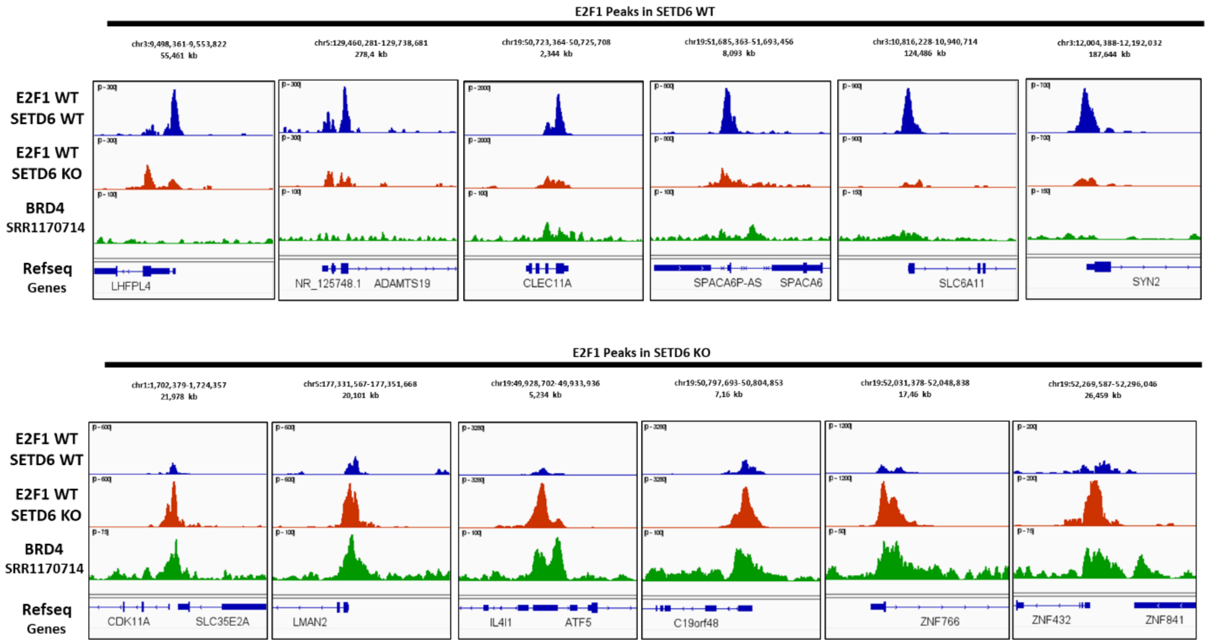

a

Supplementary Figure 12: Exemplary browser views showing co-occurrence of BRD4 and E2F1 peaks in SETD6 KO cells, but only E2F1 peaks in SETD6 WT cells. The figure was prepared using IGV 2.13.1, ChIP-seq profiles for BRD4 (SRR1170714, green) and E2F1 in SETD6 WT (blue) and KO (red) cells samples are visualized.

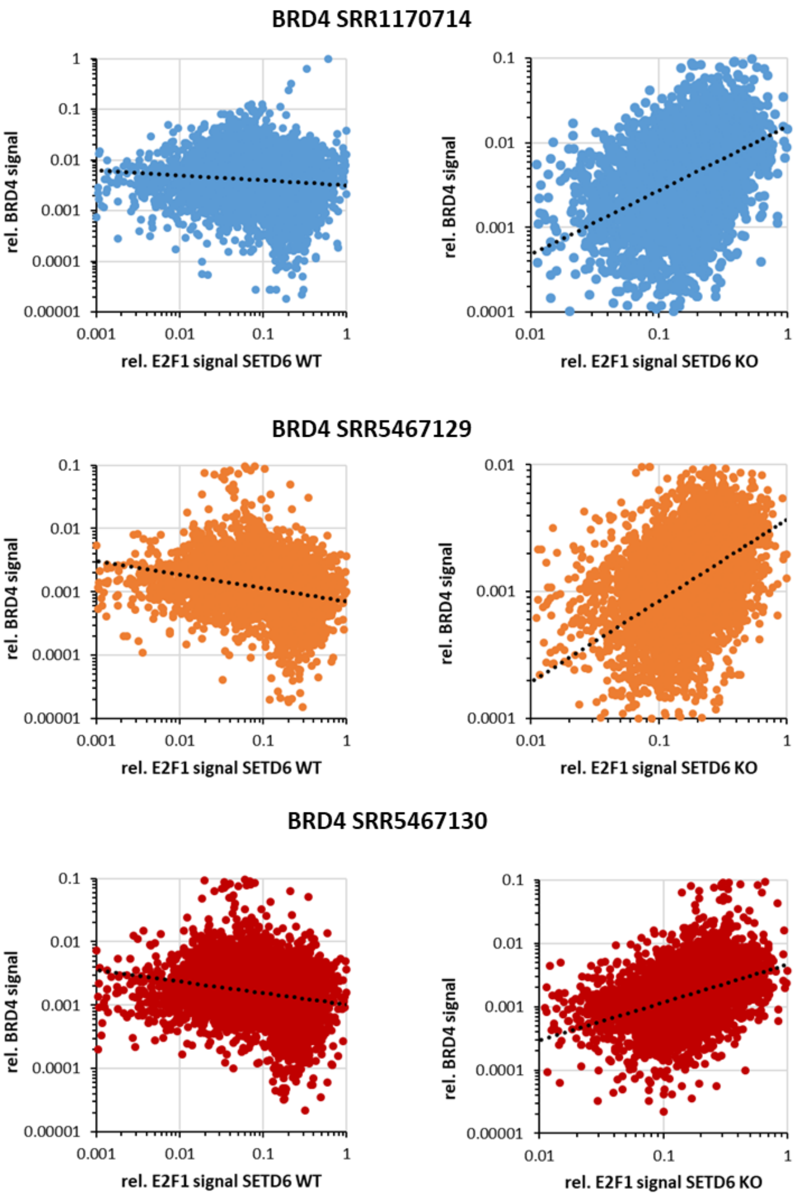

Supplementary Figure 13: Correlation analysis of BRD4 and E2F1 binding in SETD6 WT and KO cells. E2F1 and BRD4 signals were determined at E2F1 peaks and its correlation was determined. For BRD4 ChIP-seq published datasets were used SRR1170714 (blue, also shown in Figure 7C) SRR5467129 (orange), and SRR5467130 (red). The correlation factors are provided in Figure 7D.

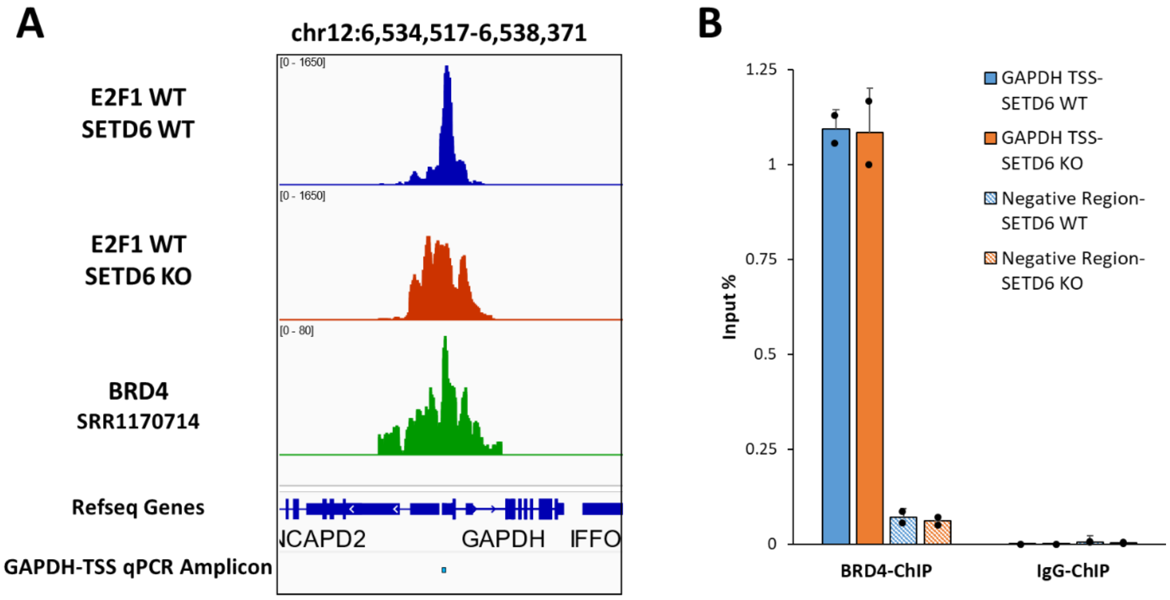

Supplementary Figure 14: Quality control of the BRD4 ChIP shown in Figure 8C. A) Browser views of the GAPDH locus showing BRD4 and E2F1 ChIP-seq peaks in SETD6 WT and KO cells, along with the GAPDH-TSS qPCR amplicon region designed for ChIP validation. **B)** The ChIP-qPCR experiment was performed by using a BRD4 specific antibody to precipitate BRD4 from sonicated chromatin. For normalization, all fold enrichment values were determined by input chromatin controls, with input percentages of 10%, 2%, and 0.4%. As negative control, an IgG antibody was used as to ensure specificity and enrichment of the immunoprecipitation. The GAPDH TSS loci (see panel A) served as positive controls for BRD4 binding while an intergenic region was used as a negative control for BRD4-binding (NCR) (taken from (3)).

Supplementary Table 1: Primers used in ChIP-qPCR assays.

Primer name	Primer sequence	Genomic region (hg38)
E2F1 forward primer	5´-GCCAAATCCTTTTTGCCGC-3`	Chr 20: 33686400-33686503
E2F1 reverse primer	5´-TAAAGCCAATAGGAACCGCC-3`	
E2F8 forward primer	5´-AGGACAACAATTCCCGACCA-3`	Chr 11: 19241041-19241134
E2F8 reverse primer	5´-AAATCGAAGGCGGAAGGTCC-3`	
Intergenic region close to CALML5 forward primer	5´- GAATGGGAGTTGGGAGAGCT-3`	Chr 10: 5499704-5499768
Intergenic region close to CALML5 reverse primer	5´- CATCCGATACCCCGACCCCAT-3`	
GAPDH TSS forward primer	5´-TCGCTCTCTGCTCCTCCTGT-3`	Chr 12: 6534515-6534591
GAPDH TSS reverse primer	5´-GTTTCTCTCCGCCCGTCTTC-3`	
Negative region forward primer	5´-TGCTGTTACTTTTTACAGGGAGTT-3`	Chr 12: 61273938-6127405
Negative region reverse primer	5´-TTTGAGCAAAATGTTGAAAACAA-3`	
MYC-p150 promoter forward primer	5´-GGACCCGCTTCTCTGAAAGG-3`	Chr 8: 127736550-127736645
MYC-p150 promoter reverse primer	5´-GCAAGTGGACTTCGGTGCTTACC-3`	
RPL21 promoter forward primer	5´-GGCCTCAGAGGTCGTTCATT-3`	Chr 13: 27251646-27251729
RPL21 promoter reverse primer	5´-ACATGGTTTAACCCGCCCAT-3`	
RPL38 promoter forward primer	5´-CGATATTTCGGGGGAGAGCG-3`	Chr 17: 74203836-74203920
RPL38 promoter reverse primer	5´-GACCTGCGGGAAACAGTCC-3`	
C6orf226 promoter forward primer	5´-GAGTTCCTGGCCCTGCTG-3`	Chr 6: 42890686-42890837
C6orf226 promoter reverse primer	5´-AAGTGCTCGCTTCTCTGCA-3`	

TBCC Promoter forward primer	5´-CGCTTTTGTTTCCGCCTTTC -3`	Chr6:42745936-42746121
TBCC Promoter reverse primer	5´-CGGAGGAAGGAAGACAAGAGA -3`	

Supplementary Table 2: Primers used in RT-qPCR assays.

**Primer name**	**Primer sequence**
C6orf226 forward primer	5´-ATCCACGGCGAACCCACAGCG -3`
C6orf226 reverse primer	5´-CGCAGTCGCTGCCTCAACCAA -3`
TBCC forward primer	5´-AACTCGCTGGCTCTCTTCTC -3`
TBCC reverse primer	5´- GGAAAGGATGCTGCTTCGTC-3`
RPL21 forward primer	5´- GGTACCTGGGTTCAACTAAAGC -3`
RPL21 reverse primer	5´- CATAGGGAATAGGTTCCAGCA -3`
RPL38 forward primer	5´- GACGAAAGGATGCCAAATCT -3`
RPL38 reverse primer	5´- GTCAGTGATGACCAGGGTGTAA -3`
MYC forward primer	5´-CTCTGACCTTTTGCCAGGAG -3`
MYC reverse primer	5´-CCTACCCTCTCAACGACAGC -3`
GAPDH forward primer	5´-AGCCACATCGCTCAGACAC-3`
GAPDH reverse primer	5´-GCCCAATACGACCAAATCC-3`

Supplementary Table 3: List of the 40 genes strongly bound and upregulated by E2F1 WT but not E2F1 E117K in WT SETD6 DU145 cells.

ABHD4	KCTD1
AGFG1	LDB1
ARRDC2	LGR4
ASNA1	LTBP1
BHLHE40	NSF
BMP4	OSGIN2
CCL28	PCP2
CDH1	PMEPA1
ERMARD	PPM1L
FAM83B	PRTG
FKBP7	RHOU
FOXA1	RNF217
FOXRED2	SIM2
FSTL4	SNTB1
GFER	TGFB3
GLS	TNFRSF13C
HOXA11-AS	TTC9
HOXA13	WDR17
JAG1	YPEL2
JAG2	ZNF503-AS1

Supplementary Table 4: List of the 210 genes strongly bound and upregulated by E2F1 WT but not E2F1 E117K in KO SETD6 DU145 cells.

AASS
ABCA2
ACTRT3
ADRB2
AKT1S1
ALDH3B1
ALG1
ALOXE3
AP5Z1
ARHGEF1
ARL6IP6
ASB7
ATP13A2
BCAR1
BCKDK
BTC
C16orf58
C16orf95
C17orf100
C19orf66
C21orf33
C21orf59
C6orf226
C8orf33
CAAP1
CAMKMT
CCDC124
CCDC97
CD70
CDC20
CES2
CHFR
CHRNB1
CNOT3
COL6A1
COX10
COX18
CRB3
CSK
CYP27A1
CYP2S1
DCAF12
DCUN1D5
DDIT4
DDRGK1
DEDD2
DHX37
DNAJB11
DNAJC2
DUSP6
DVL2
EFEMP2
ELK1
EME2
ETAA1
EXOSC3
EXOSC4
FAHD2B
FAM131A
FAM216A
FBXW5
FBXW9
FLNC
G0S2
GLS2
GPX1
GRWD1
HLA-H
HM13
HOXB5
HSPA4
HSPBP1
INTS3
ISCA2
ISCU
ITGA7
ITSN1
KAT7
KIAA1468
KLHL7
KLHL9
KTI12
L3MBTL1
LBX2
LINC00482
LINC01023
LSR
MAD1L1
MAN2B1
MAP3K6
MAP3K7
MED15
MED17
MFSD1
MIS18A
MOCS3
MOSPD3
MRM1
MRPL12
MRPL34
MRPL37
NARS2
NCDN
NDUFS3
NFATC2
NGRN
NR1H2
NUDT16
NUMA1
NUP133
OIP5
OSR1
PAPOLA
PARL
PARP10
PCDHA3
PFAS
PGAP3
PHB2
PHLDB3
PIP5KL1
PLEKHG5
PLEKHN1
POLR2A
PPP1R15A
PRADC1
PRCC
PRDM15
PRKAR1B
PRKD2
PTK6
PTPN23
QPCTL
QRFPR
RAB34
RAVER1
RHOD
RNF126
RPRD1B
RPS19BP1
RPUSD2
RRP9
RRS1
RTKN
SAMD14
SBDSP1
SEC16A
SEMA6A
SFSWAP
SHKBP1
SIRT6
SIX5
SLC25A3
SLC25A46
SLCO4A1-AS1
SMTN
SNRPA
SORBS3
SPATA2
SPHK2
SSH3
STARD10
STMN3
TAF1C
TBC1D2
TBC1D22A
TBCC
TESK1
TFE3
TICAM1
TIMM44
TM9SF2
TMEM115
TMEM143
TMEM218
TMEM70
TMPO-AS1
TNKS1BP1
TOPORS
TRAPPC6A
TRIM35
TRIP6
TRMT2B
TSC22D4
TUBB3
TUFM
UBE2S
UCKL1-AS1
UNC13D
USP18
VPS18
VPS9D1
VPS9D1-AS1
WDR18
WDR24
YARS2
YOD1
YTHDF3-AS1
ZEB1-AS1
ZFPL1
ZNF287
ZNF408
ZNF419
ZNF526
ZNF581
ZNF582
ZNF593
ZNF646
ZNF668
ZNF773

Supplementary references

1. Kublanovsky, M., Ulu, G.T., Weirich, S., Levy, N., Feldman, M., Jeltsch, A. and Levy, D. (2023) Methylation of the transcription factor E2F1 by SETD6 regulates SETD6 expression via a positive feedback mechanism. *J Biol Chem*, **299**, 105236.
2. Vershinin, Z., Feldman, M., Werner, T., Weil, L.E., Kublanovsky, M., Abaev-Schneiderman, E., Sklarz, M., Lam, E.Y.N., Alasad, K., Picaud, S. *et al.* (2021) BRD4 methylation by the methyltransferase SETD6 regulates selective transcription to control mRNA translation. *Sci Adv*, **7**.
3. Mio, C., Lavarone, E., Conzatti, K., Baldan, F., Toffoletto, B., Puppin, C., Filetti, S., Durante, C., Russo, D., Orlacchio, A. *et al.* (2016) MCM5 as a target of BET inhibitors in thyroid cancer cells. *Endocr Relat Cancer*, **23**, 335-347.
